# Novel insights into the pathogenicity of epidemic *Aeromonas hydrophila* ST251 clones from comparative genomics

**DOI:** 10.1038/srep09833

**Published:** 2015-05-27

**Authors:** Maoda Pang, Jingwei Jiang, Xing Xie, Yafeng Wu, Yuhao Dong, Amy H. Y. Kwok, Wei Zhang, Huochun Yao, Chengping Lu, Frederick C. Leung, Yongjie Liu

**Affiliations:** 1 College of Veterinary Medicine, Nanjing Agricultural University, Nanjing, 210095, China; 2 Bioinformatics Center, Nanjing Agricultural University, Nanjing, 210095, China; 3 School of Biological Sciences, University of Hong Kong, Hong Kong SAR, 999077, China

## Abstract

Outbreaks in fish of motile *Aeromonad* septicemia (MAS) caused by *Aeromonas hydrophila* have caused a great concern worldwide. Here, for the first time, we provide two complete genomes of epidemic *A. hydrophila* strains isolated in China. To gain an insight into the pathogenicity of epidemic *A. hydrophila*, we performed comparative genomic analyses of five epidemic strains belonging to sequence type (ST) 251, together with the environmental strain ATCC 7966^T^. We found that the known virulence factors, including a type III secretion system, a type VI secretion system and lateral flagella, are not required for the high virulence of the ST251 clonal group. Additionally, our work identifies three utilization pathways for *myo*-inositol, sialic acid and L-fucose providing clues regarding the factors that underlie the epidemic and virulent nature of ST251 *A. hydrophila*. Based on the geographical distribution and biological resources of the ST251 clonal group, we conclude that ST251 is a high-risk clonal group of *A. hydrophila* which may be responsible for the MAS outbreaks in China and the southeastern United States.

*Aeromonas hydrophila* is ubiquitous in various aquatic environments, and has been considered as a pathogen of fish, amphibians, reptiles and mammals^1^. Being an opportunistic pathogen, *A. hydrophila* could still cause the outbreaks of motile *Aeromonad* septicemia (MAS) in fish. As has been reported, MAS has frequently caused huge economic losses in the cyprinid fish industry throughout China since 1989^2^. From the summer of 2009, MAS outbreaks have also occurred in the southeastern United States, resulting in industry-wide losses of channel catfish^3^,^4^. This disease has become an increasingly prominent problem for the rapid development of aquaculture industry.

The pathogenicity of *A. hydrophila* is usually considered to be multifactorial. Over the last 30 years, a number of virulence factors, including secretion systems, motility and adhesins, toxins, enzymes, quorum systems, iron acquisition and antibiotic resistance, have been identified^1^. To date, most of the research on *A. hydrophila* has still focused on the so-called virulence factors. However, recently, increasing reports^5^,^6^,^7^ have proposed that animal environments that pathogens colonize have likely driven the evolution of new metabolic adaptations to maximize these new nutritional opportunities, and these adaptations may link with bacterial virulence. These suggest the known virulence factors may not be the only players in the bacterial infection process.

In this study, we present the first two complete genomes of epidemic *A. hydrophila* strains isolated in China, NJ-35^8^ and J-1^9^, which were responsible for the MAS outbreaks in Jiangsu Province, in 2010 and 1989, respectively. To address the causes of the epidemic and highly virulent properties of *A. hydrophila*, and to clarify the evolution relationships among the epidemic strains isolated from China and the United States, we performed comparative genomic analyses of five epidemic strains isolated from both countries, together with the environmental strain ATCC 7966^T^. Based on comparative genomic and functional analyses, we provide novel insights into the pathogenicity of epidemic *A. hydrophila* by identifying putative factors that may be involved in the bacterial pathogenicity, and we also determined that the ST251 clonal group of *A. hydrophila* is likely responsible for the ongoing MAS outbreaks in China and the southeastern United States.

## Results

### General features of six *A. hydrophila* genomes

**Figure 1:**
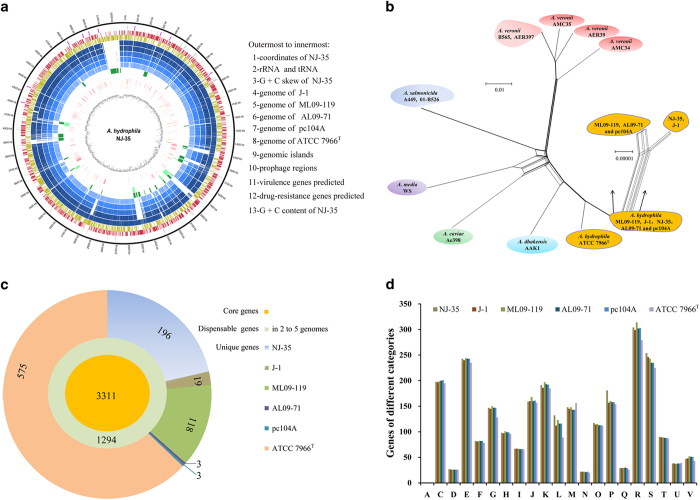
(**a**) Circular representation of six *A. hydrophila* genomes. The genomes were aligned with *dnaA* in the initial position and sequences moving clockwise. (**b**) Phylogenetic network of *A. hydrophila* and closely related *Aeromonas* species. Strains of the same species are displayed using identical colors. (**c**) Core genes and dispensable genes of six *A. hydrophila* genomes. (**d**) Comparison of COG categories among six *A. hydrophila* genomes. The ordinate axis indicates the number of genes in each COG functional category assigned to each of the six *A. hydrophila* genomes.

The genomes of *A. hydrophila* strains NJ-35 and J-1 were sequenced and submitted to GenBank (http://www.ncbi.nlm.nih.gov/Genbank/index.html). A whole-genome overview is shown in Table 1 and Fig. 1(a). Among the six strains studied, *A. hydrophila* NJ-35^8^, J-1^9^, ML09-119^3^,^10^ and AL09-71^4^,^11^,^12^ were epidemic piscine strains, whereas ATCC 7966^T^^13^ was an environmental strain whose genome has been investigated extensively. Strain pc104A isolated from the soil of a catfish pond was from western Alabama, which has experienced an epidemic outbreak of MAS since 2009^14^. The genomes of six strains comprised a single circular chromosome which range in size from 4.74 Mb to 5.28 Mb. The G + C% content of each genome ranged from 60.51% to 61.50%, and the number of predicted CDS varied from 4119 to 4526. There were 10 rRNA operons encoding 31 rRNAs in each genome. Unlike rRNAs, the number of tRNAs in strain ATCC 7966^T^ was obviously higher than in the other five strains, and the increasing number of tRNAs may be useful for increasing the rate of protein synthesis^15^.

### Virulence factors in *A. hydrophila*

In previous studies, strains NJ-35^8^, J-1^9^, ML09-119^3^,^10^ and AL09-71^4^,^11^,^12^ were reported to be highly virulent strains, and pc104A^14^ was a moderately virulent strain, whereas the virulence of the environmental strain ATCC 7966^T^ was found to be significantly lower than those of the clinical isolates^16^. To determine which known virulence factors may contribute to the pathogenicity of the epidemic strains, we listed nearly all of the known virulence factors and queried them in six *A. hydrophila* genomes (Table 2). Surprisingly, genes encoding all the known virulence factors, with the exception of type III secretion system (T3SS), type VI secretion system (T6SS) and lateral flagella, were present in each *A. hydrophila* genome. T3SS and lateral flagella were absent not only in the environmental strain but also in the epidemic strains, whereas T6SS was only absent in the American strains ML09-119, AL09-71 and pc104A.

Moreover, to identify whether there were additional antibiotic resistance genes present in epidemic strains, each genome were annotated based on the Antibiotic Resistance Genes Database (ARDB, version 1.1; http://ardb.cbcb.umd.edu/), and 23 potential drug-resistance types were identified (Table S2). Although the six strains were isolated in different locations and at different times, antibiotic resistance genes were distributed similarly among these strains. The only exception is that a gene encoding aminoglycoside resistance has been not found in American strains ML09-119, AL09-71 and pc104A. While these *A. hydrophila* strains did not exhibit all drug-resistance phenotypes, the potential for antibiotic resistance in *A. hydrophila* merits increased attention.

Finally, to rule out the possibility that some virulence factors were not queried due to a careless omission, we also annotated the six *A. hydrophila* genomes using the Virulence Factors Database (VFDB, updated: 2012; http://www.mgc.ac.cn/VFs/v3index.htm). Intriguingly, the virulent strains NJ-35, J-1, ML09-119, AL09-71 and pc104A contained 193, 193, 191, 181 and 181 genes, respectively, which were less than ATCC 7966^T^ (195 genes). However, most dispensable virulence genes were located in O-antigen gene clusters and T6SS, and there were few findings except for an LPS-modification gene cluster specific to strain ATCC 7966^T^ (Dataset S1).

The above analyses showed that the known virulence factors had similar distributions among the epidemic strains and the environmental strain. This finding raised the question of whether the six *A. hydrophila* strains really differed in virulence. Thus, we reassessed the virulence of these strains using zebrafish and ICR mice. Because strain ML09-119, AL09-71 and pc104A was absent in our laboratory, their virulence was not assessed. As shown in Fig. S1a, the LD_50_s in zebrafish of strains NJ-35, J-1 and ATCC 7966^T^ were 1.30 × 10^2^, 1.94 × 10^3^, and 1.05 × 10^6^ CFU/fish, respectively. According to our previous study^8^, NJ-35 and J-1 could be classified as virulent strains (LD_50_ < 1.0 × 10^6^CFU/fish), whereas ATCC 7966^T^ is better classified as an avirulent strain (LD_50_ > 1.0 × 10^6^CFU/fish). Similarly, the virulence of strains NJ-35 and J-1 were also significantly greater than that of ATCC 7966^T^ in ICR mice (Fig. S1b). Therefore, we speculated that there might be undiscovered virulence factors that play key roles in the pathogenicity of *A. hydrophila*.

### Whole-genome comparisons

#### Phylogenetic network analysis

To determine the evolutionary relationships among the six *A. hydrophila* genomes, a phylogenetic network was constructed using 905 concatenated genes with a total length of 1.01 Mb. Ten genomes from other *Aeromonas* species were used as references. As shown in Fig. 1b, unlike strain ATCC 7966^T^, the other five virulent *A. hyrophila* strains fell into the same subclade. Further, the two Chinese epidemic strains (NJ-35 and J-1) clustered together, whereas the three American strains (ML09-119, AL09-71 and pc104A) formed a separate linage. In addition, we calculated the average nucleotide identity (ANI) using JSpecies (version 1.2.1; http://imedea.uib-csic.es/jspecies/about.html). As shown in Table S3, the ANI values among the five virulent strains ranged between 99.81–100%, whereas the values between them and ATCC 7966^T^ ranged between 96.74–96.79%. These results suggested that strain pc104A should belong to the clonal group of epidemic *A. hydrophila* isolates which caused large-scale MAS outbreaks in the United States. The five epidemic strains were more similar to each other than to strain ATCC 7966^T^, indicating that some genetic traits distinctive to the environmental strains may appear in the epidemic strains.

#### Core and dispensable genomes

To find the essence and diversity of the six *A. hydrophila* strains, we analyzed their core and dispensable genomes. The pan-genome shared by the six strains consisted of 5519 genes, including 3311 core genes and 2208 dispensable genes consisting of 914 unique genes and another 1294 genes present in two to five strains (Fig. 1c). We found that ATCC 7966^T^ has 575 unique genes, while epidemic strains NJ-35, J-1, ML09-119, AL09-71 and pc104A have 196, 19, 118, 3 and 3 unique genes, respectively. The smaller amount of unique genes in strains J-1, AL09-71 and pc104A occurs because most of their genes are contained by NJ-35 or ML09-119. The diversity among the six genomes provides genetic clues about the mechanisms contributing to bacterial virulence. However, relatively high proportions of dispensable genes, especially unique genes, are predicted to encode hypothetical proteins, which create challenges for further analysis.

#### COG functional categories

To identify which function of the epidemic strains may lead to the virulence diversity, we annotated the six *A. hydrophila* genomes using the protein database of Clusters of Orthologous Groups (COGs, updated: 2014; http://www.ncbi.nlm.nih.gov/COG/). As depicted in Fig. 1d, the gene numbers of the five epidemic strains categorized as COG G, L, P, R and S were relatively more than that of strain ATCC 7966^T^. This finding suggested that the genes responsible for various functions, such as carbohydrate metabolism and transport (COG G), replication, recombination and repair (COG L), inorganic ion transport and metabolism (COG P) and poorly characterized function (COG R and S), may contribute to the diversity in bacterial pathogenicity. To further analyze the genes causing the various gene numbers of COG G, L, P, R and S, we aligned these genes using strain NJ-35 as the reference (Dataset S2). Considering that COGs R and S were poorly characterized, we mainly focused on the genes within COGs G, L and P. Intriguingly, among the genes classified as COG G, some genes involved in the utilization of *myo*-inositol, sialic acid and L-fucose were found to be specific to all five epidemic strains. Among the COG L genes, plentiful genes encoding integrase and transposase were present in the five epidemic strains but absent in ATCC 7966^T^. Their presence suggested that more HGT (horizontal gene transfer) or transposition may have occurred in epidemic strains. Among the COG P genes, the accumulation of ABC-type transporters was an important factor affecting the number of genes categorized as COG P genes in strain NJ-35.

### Regions of genomic plasticity among the *A. hydrophila* strains

To identify the pathogenicity-related regions, we identified 27 regions of genomic plasticity (RGPs). Of the 27 RGPs, 24 were predicted to be genomic islands using IslandViewer software (updated Sep 3, 2014; http://pathogenomics.sfu.ca/islandviewer). The epidemic strains NJ-35, J-1, ML09-119, AL09-71 and pc104A contained 18, 13, 17, 17 and 17 RGPs, respectively, which were significantly more than ATCC 7966^T^ (6 RGPs). By analyzing the gene content of the 27 RGPs, we identified three O-antigen regions, 10 prophage regions and 15 other functional RGPs (Table 3, Dataset S3).

#### (I)O-antigen regions

**Figure 2:**
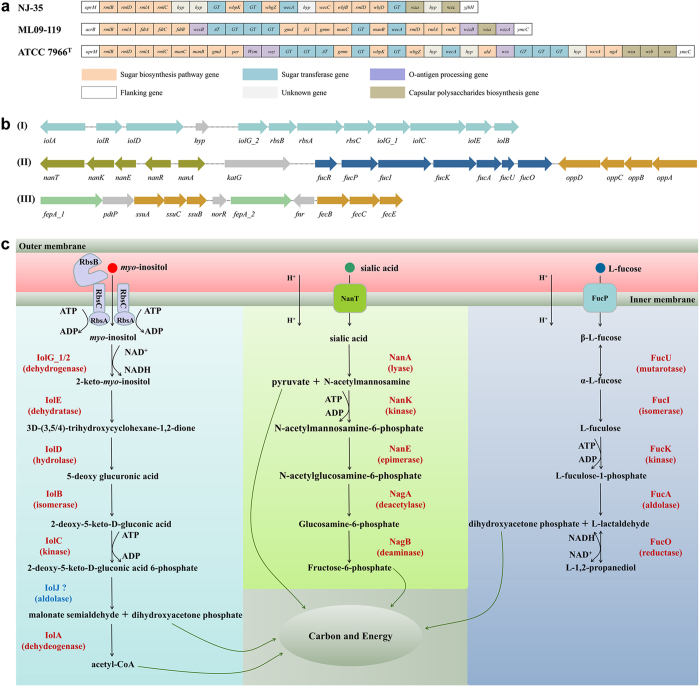
(**a**) Genetic organization of the O-antigen gene clusters of *A. hydrophila* strains. *GT* represents the glycosyltransferase gene, *AT* represents the acetyltransferase gene, *hyp* represents the gene encoding the hypothetical protein. (**b**) Genetic organization of partial gene clusters of RGP3, RGP13 and RGP15. (I) depicts the *myo*-inositol utilization gene clusters; (II) depicts the sialic acid and L-fucose utilization gene clusters and *oppABCD* gene clusters; and (III) depicts the *ssuABC* and *fecBCE* gene clusters. *Hyp* represents the gene encoding the hypothetical protein. (**c**) Proposed utilization pathways for *myo*-inositol, sialic acid and L-fucose in *A. hydrophila*. The three proposed utilization pathways were modified according to previous studies^18^,^19^,^26^ and the genome annotation in KEGG pathway database. IolJ was not identified in *A. hydrophila*.

RGP1, RGP21 and RGP26 carry diverse O-antigen gene clusters (Fig. 2a, Table 3, Table S4). The O-antigen gene clusters in strains NJ-35 and J-1 were identical and have not been observed previously in *A. hydrophila*, while strains ML09-119, AL09-71 and pc104A share the identical O-antigen gene clusters. All six O-antigen gene clusters contained the genes *rmlA*, *rmlB*, *rmlC* and *rmlD,* which encode proteins responsible for the biosynthesis of dTDP-L-rhamnose from glucose-1-phosphate. However, the organization and homology of the genes of the unique O-antigen gene cluster type varied substantially. In strains ML09-119, AL09-71, pc104A and ATCC 7966^T^, the *manB* and *manC* genes were located in O-antigen gene clusters, whereas *manA* was found elsewhere in the genomes. These three genes are required for the synthesis of GDP-mannose from fructose-6-phosphate^10^; however, they were not found in strains NJ-35 and J-1. In addition, *fdtA*, *fdtB* and *fdtC* which are required for the synthesis of the dTDP-sugar 3-acetamido-3,6-dideoxy-D-galactose^10^, were found to be specific to strains ML09-119, AL09-71 and pc104A.

#### (II) Prophage regions

A total of eight prophage regions (prophage−1 to prophage−8) were identified by PHAST (updated Sep. 30, 2014; http://phast.wishartlab.com); their features are listed in Table S5, S6 and Fig. S2. Prophage−9 and prophage−10 were found in strain ATCC 7966^T^ in previous studies^13^,^16^, although they were not identified by PHAST. Among the 10 known prophages, prophage−3, −6 and −8 were predicted to be intact prophages. Interestingly, all of the prophages identified in the five epidemic strains were absent in strain ATCC 7966^T^, and prophages−1, −4, −6 and −7 were conserved in all five epidemic strains. Prophages are thought to be important for bacteria because they often confer fitness and/or virulence factors. However, our analyses showed that none of the known virulence factors are present in the prophage regions (Fig. 1a). Nevertheless, the vast number of hypothetical proteins contained in the prophage regions may play roles in the evolution and virulence of *A. hydrophila*, and they therefore merit further functional characterization.

#### (III) Other RGPs

In addition to the O-antigen and prophage regions, 15 other RGPs were analyzed (Table 3). RGP2, RGP6, RGP8, RGP14, and RGP20 were classified as unknown RGPs because most of the proteins encoded by these regions were predicted to be hypothetical proteins and their functions were poorly understood. RGP7, RGP11, and RGP19 were classified as uncharacterized RGPs because the functions of some of their genes had been predicted but no representative gene clusters had been identified. The remaining seven RGPs were relatively well characterized or contained representative gene clusters. RGP10 consisted of a T6SS gene cluster that was absent in the three American strains. RGP13 contained two gene clusters encoding sialic acid and L-fucose utilization pathways. RGP15 contained two ABC-type transport systems, SsuACB and FecBCE. RGP17 also contained an ABC-type transport system, which is involved in the transport of DL-methionine. RGP18 specific to strain NJ-35 was an integrative plasmid containing 140 genes. This region was found to contain numerous genes involved in DNA replication, transcription and repair. However, no virulence factors were observed in this integrative plasmid. RGP22 specific to American epidemic strains contained the *torCAD* operon encoding a trimethylamine N-oxide (TMAO) reductase respiratory system^17^. RGP27 specific to ATCC 7966^T^ consisted of abundant genes involved in LPS modification. In addition, a *myo*-inositol utilization pathway was observed adjacent to prophage−1 in RGP3.

#### (IV) RGPs specific to epidemic strains

To elucidate the key determinants of pathogenicity of *A. hydrophila*, we focused on the RGPs that were specific to the five epidemic strains. Among these RGPs, only RGP3, RGP13, and RGP15 were characterized and therefore further analysed using strain NJ-35 as the reference (see more details in Table S7).

The gene clusters in RGP3 encoded 11 proteins that were associated with the *myo*-inositol utilization pathway (Fig. 2b). As shown in Fig. 2c, once *myo*-inositol would be transported into the cytoplasm, *myo*-inositol 2-dehydrogenase (IolG_1/IolG_2) would initiate the degradation of *myo*-inositol, after which IolEDBCJA would be required for the degradation step. The net result of the inositol metabolic pathway in *A. hydrophila*, thus, would be the conversion of *myo*-inositol to a mixture of dihydroxyacetone phosphate and acetyl-CoA. Dihydroxyacetone phosphate can enter the glycolysis metabolic pathway, while acetyl-CoA conveys carbon atoms within the acetyl group to the citric acid cycle, to be oxidized for energy production. Similarly to the findings of a previous study^10^, IolJ (2-deoxy-5-keto-D-gluconic acid 6-phosphate aldolase was not identified in each *A. hydrophila* genome. However, other aldolases, such as fructose/tagatose bisphosphate aldolase (U876_19685), which shares similar amino acid sequences with IolJ, could potentially be a substitute for IolJ.

RGP13 contained gene clusters encoding two metabolic pathways in which sialic acid and L-fucose are used (Fig. 2b). The sialic acid utilization pathway was encoded by the *nanTKERA* operon and other two genes*—nagA* and *nagB*—which are located outside of RGP13 (Fig. 2c). Briefly, sialic acid from the surrounding environment is transported into the cytoplasm by a symporter (NanT) through the inner membrane. A pyruvate group is first removed from the sialic acid with the help of N-acetylneuraminate lyase (NanA), after which N-acetylmannosamine is catalyzed by NanK, NanE, NagA and NagB, thereby generating fructose-6-phosphate, which may then enter the glycolysis metabolic pathway^18^. The pyruvate produced in this process is at a crossroads of central metabolic pathways, and it can yield acetyl-CoA to provide energy under aerobic conditions. The L-fucose utilization pathway was encoded by the *fucRPIKAUO* operon in the same orientation (Fig. 2b). L-fucose is transported into cytoplasm by a symporter (FucP); L-fucose isomerase (FucI) then converts α-L-fucose to L-fuculose with the help of fucose mutarotase (FucU), which can catalyze natural mutarotation between α-L-fucose and β-L-fucose. Ultimately, L-fucose can be degraded into dihydroxyacetone phosphate, which lies in the glycolysis metabolic pathway, and L-1,2-propanediol, which is often secreted from cells^19^ (Fig. 2c). Furthermore, a proposed ABC-type dipeptide/oligopeptide/nickel transport systems (OppABCD) was identified (Fig. 2b).

In RGP15, another two ABC-type transport systems were also identified (Fig. 2b), one of which was encoded by *ssuACB* and predicted to transport nitrate/sulfonate/bicarbonate and the other of which was encoded by *fecBCE* and predicted to transport Fe^3+^–siderophores. In addition, two TonB-dependent receptors (FecA_1 and FecA_2) were identified near the transport systems. These ABC-type transport systems may contribute to the fitness of *A. hydrophila* by acquiring essential ions or nutrients efficiently in a nutrient-limiting host environment.

### MLST analysis and the distribution of putative virulence factors

**Figure 3:**
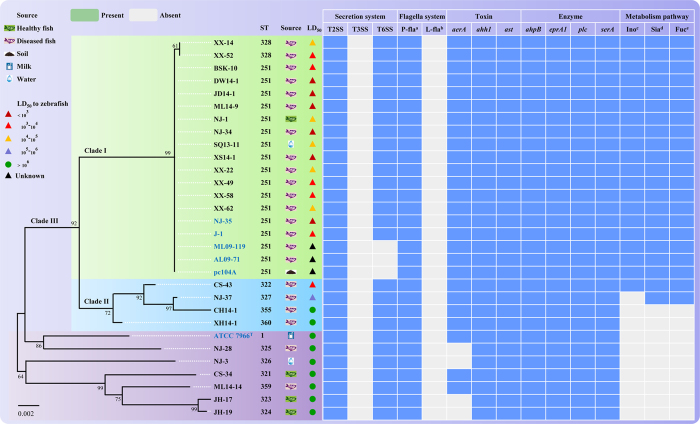
MLST analysis and the distribution of putative virulence factors in *A. hydrophila*. ^a,b^P-fla and L-fla represent the polar flagellum and lateral flagella, respectively; ^c,d,e^Ino, Sia and Fuc represent the utilization pathways for *myo*-inositol, sialic acid and L-fucose, respectively.

To determine whether there exists a link among ST, virulence factors and bacterial virulence, we performed the multilocus sequence typing (MLST) of 30 *A. hydrophila* strains, including the six *A. hydrophila* strains analyzed and another 24 strains with known virulence, as determined in our previous study^8^ and in this study (Fig. S3). A total of 13 STs were identified in the 30 strains, and 17 virulent strains including the five epidemic strains analyzed all belonged to ST251 (Fig. 3). ST251 and ST328 belonged to the same clonal group (ST251 clonal group) because they shared five alleles out among the six housekeeping genes^20^ (*gyrB*, *groL*, *gltA, metG*, *ppsA* and *recA*). We also constructed a phylogenetic tree based on the six housekeeping genes. As shown in Fig. 3, the strains analyzed in the phylogenetic tree could be classified into clade I (ST251 and ST328), clade II (ST322, ST327, ST355 and ST360) and clade III (ST1, ST321, ST323, ST324, ST325, ST326 and ST359). Intriguingly, a high correlation between the genetic phylogeny and LD_50_s of *A. hydrophila* strains in zebrafish was observed. All strains placed in clades I could be classified as virulent strains (LD_50_ < 1.0 × 10^6^CFU/fish), whereas strains placed in clade III were all considered avirulent strains (LD_50_ > 1.0 × 10^6^CFU/fish).

The most important finding was the distribution of virulent factors among *A. hydrophila* strains. With respect to the secretion systems, type II secretion system (T2SS) was observed in all 30 strains, T6SS was observed in 27 strains (all strains except the three American epidemic strains), and T3SS was not observed in any strain. A polar flagellum was present in each strain, whereas lateral flagella were absent in all strains. Among the detected genes encoding toxins and enzymes, only *aerA* was absent in all four avirulent strains; the other genes were all present in both virulent and avirulent strains. Intriguingly, the three metabolic pathways appeared only in strains belonging to the ST251 clonal group, ST322 and ST327, and all of these strains were virulent. Moreover, the ATCC 7966^T^, CH-14-1, XH14-1, ML14-14 and CS-34 strains, which lack the three metabolic pathways found in the virulent strains, were avirulent strains, even though they contained *aerA*. These findings suggested that the utilization pathways for *myo*-inositol, sialic acid, and L-fucose may be linked with the full virulence of epidemic *A. hydrophila*.

### The utilization of myo-inositol, sialic acid and L-fucose

**Figure 4:**
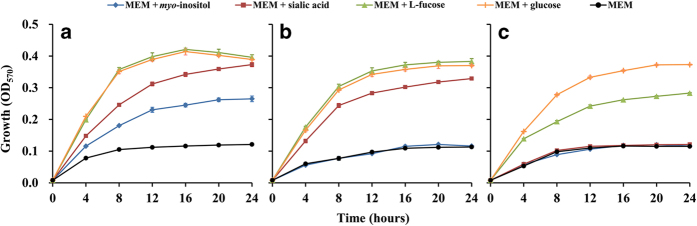
Growth of *A. hydrophila* strains in MEM supplemented with different chemicals. The growth of strains NJ-35 (**a**), NJ-37 (**b**) and ATCC 7966^T^ (**c**) is depicted.

All strains containing the *myo*-inositol utilization, sialic acid and L-fucose utilization pathways could utilize the corresponding chemicals as carbon sources for growth. However, the level of bacterial growth on the different chemicals was diverse. As shown in Fig. 4, the growth of strains NJ-35, NJ-37 and ATCC 7966^T^ was examined in comparison with bacterial growth on glucose. The growth of strain NJ-35 on L-fucose was similar to that on glucose but was faster than the growth on *myo*-inositol and sialic acid (Fig. 4a). This phenomenon was also observed in the other tested strains, suggesting that *A. hydrophila* could utilize L-fucose to grow more efficiently. As depicted in Fig. 4b, strain NJ-37, which lacks the *myo*-inositol utilization pathway, could not utilize *myo*-inositol to grow, but it could grow using sialic acid and L-fucose as carbon sources. Surprisingly, strain ATCC 7966^T^, without the predicted L-fucose utilization pathway, also had the ability to grow on L-fucose (Fig. 4c). This phenomenon was also observed in strain NJ-3, which also lacks the L-fucose utilization pathway. However, both ATCC 7966^T^ and NJ-3 grew much more slowly on L-fucose than on glucose. This result indicated that the ATCC 7966^T^ and NJ-3 strains may have an alternative metabolic pathway capable of utilizing L-fucose and that this pathway cannot utilize L-fucose as effectively as the classical L-fucose utilization pathway.

### Geographical distribution of the *A. hydrophila* ST251 clonal group

**Figure 5:**
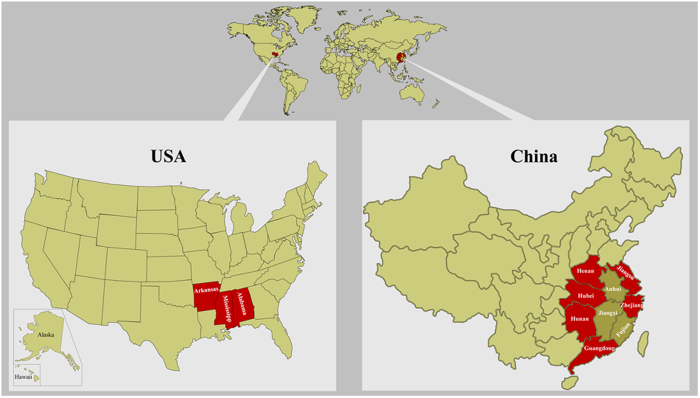
Geographical distribution of the ST251 clonal group *A. hydrophila*. The regions filled in with red represent the distribution of the ST251 clonal group. This map was modified based on the maps obtained from PowerPoint Toolkit (http://ppt-toolkit.com/).

Since all the epidemic strains analyzed were belong to ST251 clonal group, we investigated the geographical distribution of this clonal group through querying related sequences on the *Aeromonas* spp. MLST website (http://pubmlst.org/Aeromonas) and in GenBank. As summarized in Fig. 5 and Table S8, the ST251 clonal group of *A. hydrophila* has been found in the Jiangsu, Henan, Hubei, Hunan, Guangdong and Zhejiang provinces of China and in Alabama, Mississippi and Arkansas in the United States. Unfortunately, the nucleotide sequences of the six housekeeping genes of most of the isolated *A. hydrophila* strains have not been analyzed, which makes it difficult to evaluate the distribution of the ST251 clonal group satisfactorily. Considering most of the provinces in southern China including Anhui, Jiangxi and Fujian all experienced epidemic outbreaks of MAS between 1989 and 1991^2^, it is likely that the ST251 clonal group can also be found in these districts.

## Discussion

In this study, we try to clarify the factors that give *A. hydrophila* its epidemic and highly virulent nature. *A. hydrophila* possesses a variety of virulence genes, and some of them, such as the genes in T3SS and lateral flagella, are recognized as virulence markers^13^. However, the known virulence factors distributed similarly among the virulent strains and the avirulent strains examined in this study, which were queried about the roles of these factors in the pathogenicity of the epidemic strains.

Proteins in *A. hydrophila* are thought to be transported across the two membranes by either a one-step process (T3SS and T6SS) or a two-step mechanism (T2SS)^1^. T3SS has been demonstrated to be essential to the pathogenicity of *A. hydrophila* strains AH3^21^. Contrary to expectations, T3SS was not observed in any of the strains used in our study. Therefore, we speculated that T3SS may play important roles in T3SS-positive strains; however, this process is not essential to the pathogenicity of all *A. hydrophila* strains. For this reason, careful attention should be paid when T3SS is used as an indicator of virulence. T6SS was characterized as a virulence factor in *A. hydrophila* SSU^22^, however, T6SS was found in 27 out of 30 strains analyzed but was surprisingly absent in the American epidemic strains ML09-119, AL09-71 and pc104A. Recently, an increasing number of studies have proposed that although T6SS can, in rare instances, be linked to host interactions and virulence, the broader physiological importance of this system is likely to provide a defense against simple eukaryotic cells and other bacteria in the environment^23^. The functions of T6SS have been investigated extensively in relation to the pathogenicity of *A. hydrophila*; however, it would also be useful to examine its role in microbial communities, as well. Unlike T3SS and T6SS, T2SS was conserved in all *A. hydrophila* strains used in this study. In *A. hydrophila*, T2SS is essential for the translocation of multiple proteins, including aerolysin, phospholipase, proteases and DNase, from the periplasm into the extracellular milieu^24^. In general, these proteins are associated with the destruction of various tissues, thus contributing to cell damage or disease. In view of the fact that the epidemic *A. hydrophila* strains examined in this study lacked both T3SS and T6SS but were still highly virulent, we hypothesized that T2SS is both necessary and sufficient for the pathogenicity of these strains.

It has been proposed that with the possible exception of the motor genes, the polar flagellum and lateral flagella rely on entirely distinct sets of genes^25^. The appearance of the polar flagellum in all 30 strains examined suggests that the polar flagellum is conserved in *A. hydrophila*. In contrast, our finding suggests that lateral flagella may not be species-specific but may instead be strain-specific, consistent with the results of a previous study^13^. In addition, we also detected a number of genes encoding toxins and enzymes; however, six such genes, but not *aerA*, were present in each of the 30 strains examined. Therefore, although these toxins and enzymes may be essential for the full virulence of *A. hydrophila*, it would not be reliable to determine the bacterial virulence based solely on the presence of several toxins or enzymes.

Because the known virulence factors are not primarily responsible for the substantial virulence of the epidemic ST251 clonal group, we searched for new factors may involve in their pathogenicity. The result of an analysis based on COG functional categories indicated that additional genes involved in functions such as carbohydrate metabolism and transport were present in the epidemic strains. Thus, we identified three metabolic pathways, utilizing *myo*-inositol, sialic acid and L-fucose, and we determined that *A. hydrophila* strains containing specific metabolic pathways could utilize chemicals metabolized in these pathways to grow. Except for the *myo*-inositol utilization pathway, the other two were first found in *A. hydrophila*. Metabolic versatility under nutrient constraints is a key determinant for bacterial survival and proliferation in different niches and compartments. In pathogenic *Salmonella*, *Vibrio* and *Helicobacter*, it has been shown that metabolic genes are as important for a successful infection as are the classic virulence genes^6^. *myo*-Inositol is an essential component of various metabolites, such as phosphatidylinositol-based phospholipids, that are abundant in animal and plant cells^5^. The metabolism of inositol compounds is not only linked to successful plant-bacterium interactions in members of the *Rhizobiaceae* but also to the survival of the animal pathogen *Brucella* inside its host^26^. *In vivo* screening approaches have indicated that the *myo*-inositol utilization pathway plays roles in *Salmonella typhimurium* virulence in mice, pigs, chicken and calves^5^. Sialic acids constitute a family of related sugar moieties that are found throughout the body as components of glycoproteins, gangliosides, and other sialoglycoconjugates^5^. It has been demonstrated that *Escherichia coli*, *Clostridium perfringens* and *Bacteroides fragilis* are able to utilize sialic acid as a carbon and energy source via the sialic acid utilization pathway^5^. In addition, mutants of both *S. typhimurium* and *Clostridium difficile* that are unable to catabolize sialic acid exhibit impaired expansion^27^. L-fucose is one component of the carbohydrate moiety of the mucosal glycoconjugates, and it is also used by a variety of microorganisms as a carbon and energy source^28^. An earlier study demonstrated that L-fucose utilization provides *Campylobacter jejuni* with a competitive advantage and that strains lacking the capability to use fucose are attenuated *in vivo*^19^. In addition, the genomes of *Salmonella enterica*, pathogenic *E. coli* and *C. perfringens* are also equipped with an L-fucose utilization pathway^7^.

Genetic links between the new metabolic capacities and virulence factors indicate that metabolic pathways are acquired as part of the pathogen evolution toward colonizing new niches with new food sources^6^. In our study, three metabolic pathways, utilizing *myo*-inositol, sialic acid and L-fucose, were identified in RGPs predicted as genomic island. Therefore, it seemed that the genetic determinants encoding metabolic functions were acquired by HGT to expand the metabolite target spectrum of epidemic *A. hydrophila*. Furthermore, our results showed that the *A. hydrophila* strains equipped with the three metabolic pathways were all considered virulent strains and had the ability to exploit the corresponding chemicals. These data suggest that the metabolic pathways presented in epidemic *A. hydrophila* may help the bacteria to overcome nutritional limitations *in vivo*, thereby increasing their fitness during infection and being the key factors causing their epidemic. Although interesting results are emerging, thus far they can only be considered preliminary. We can not exclude the possibility that the large numbers of hypothetical proteins present in the epidemic strains may also be responsible for the bacterial virulence. To demonstrate that the genes of these metabolic pathways indeed play a role in virulence, it is necessary to construct deletion mutants for these functions and test their virulence. Further study of the deletion mutants will be the fucous in our future work.

Considering the geographical distribution and biological resources of the ST251 clonal group, we speculated this clonal group is likely to be responsible for the outbreaks of *A. hydrophila* in China and the United States. Based on phylogenetic network and ANI analyses, we found that all the five epidemic ST251 strains were highly similar to each other. Moreover, the fact that 10 RGPs were common to the five epidemic strains also corroborates their similarity. These findings suggest that five epidemic strains analyzed may share a common ancestor. The *A. hydrophila* strain J-1 was isolated in Jiangsu Province, China, in 1989 and was used as a vaccine strain in China. Additionally, strain NJ-35 was isolated in Jiangsu Province in 2010, and this strain contains almost all of the gene content of strain J-1. Therefore, it is reasonable to speculate that the NJ-35 strain may have evolved from the J-1 strain. Hossain *et al.*^3^ proposed that ST251 strains in the United States may have been introduced from China, as *A. hydrophila* ST251 strains have been identified in China and their occurrence documented since the late 1980s but this clonal group was not observed in the United States until 2004. Although we tend to agree with this opinion, more evidence is needed to support this speculation. In particular, the complete genome information of the ST251 clonal group strains isolated from both countries will likely shed light on this issue. In China, ST251 strains have primarily been isolated from carp, whereas in the United States, they have been isolated from channel catfish. This finding could be attributed to differences in the species of farmed fish in these two countries. Of note, we showed that strains of the ST251 clonal group were also highly virulent in zebrafish, which indicated that these strains may be virulent in diverse fish species. Equally important is that *A. hydrophila* ST251 strains were still being isolated from diseased fish in our most recent survey, suggesting that ST251 is a high-risk type in fish farms. Furthermore, because of the globalization of the aquaculture and ornamental fish trade industries, the ST251 clonal group could be introduced into non-epidemic districts. Therefore, more work is urgently needed to prevent further epidemic outbreaks of ST251 *A. hydrophila* infection.

In conclusion, this report reevaluated the importance of known virulence factors, identified metabolic strategies developed by the epidemic strains, and thereby provides a comprehensive understanding of the pathogenicity of *A. hydrophila*. These findings suggest that links between metabolic adaptations of *A. hydrophila* and their ability to become epidemic and virulent should definitely receive more attention. Furthermore, the *A. hydrophila* strains of the ST251 clonal group are likely to be responsible for the MAS outbreaks in both China and the southeastern United States.

### Experimental procedures

#### Ethics statement

All animal experiments were carried out according to animal welfare standards and were approved by the Ethical Committee for Animal Experiments of Nanjing Agricultural University, China.

### Strains and genomes

Six *A. hydrophila* strains NJ-35, J-1, ML09-119, AL09-71, pc104A and ATCC 7966^T^ were used in this study to perform the comparative genomic analyses (Table 1). The ten genomes (*Aeromonas veronii* B565, AER39, AER397, AMC34 and AMC35; *Aeromonas salmonicida* A449 and 01-B526; *Aeromonas dhakensis* AAK1; *Aeromonas media* WS; *Aeromonas caviae* Ae398) used to perform the phylogenetic network analysis were deposited in GenBank with accession numbers NC_015424, NZ_AGWT00000000, NZ_AGWV00000000, NZ_AGWU00000000, NZ_AGWW00000000, NC_009348, NZ_AGVO00000000, NZ_BAFL00000000, NZ_ALJZ00000000 and NZ_CACP00000000, respectively. The 24 *A. hydrophila* strains BSK-10, CS-34, CS-43, JH-17, JH-19, NJ-1, NJ-28, NJ-3, NJ-34, NJ-37, XX-14, XX-22, XX-49, XX-52, XX-58, XX-62, CH14-1, DW14-1, JD14-1, ML14-9, ML14-14, SQ13-11, XH14-1 and XS14-1 were used to examine the distribution of putative virulence genes and to perform MLST analysis. The sequences of housekeeping genes (*gyrB*, *groL*, *gltA, metG*, *ppsA* and *recA*) of the 24 strains were deposited in GenBank with accession numbers KP162270 to KP162274, KM235114 to KM235188, and KP781915 to KP781962.

### Sequencing, assembly and annotation

The genomes of NJ-35 and J-1 were sequenced using 454 GS Junior pyrosequencing with 23-fold and 29-fold coverage, respectively. One shotgun run and one 8 kb-library span paired-end run were conducted, the raw data were assembled by Newbler (version 2.7; Roche/454 Life Science) with default parameters. In addition, gap-filling primers were designed and the Sanger sequencing results were compiled with the contigs into the complete genomic sequence via SeqMan (version 7.1.0; Larsegene). Then, the complete genomes of strains NJ-35 and J-1 were obtained and annotated using the Prokaryotic Genome Automatic Annotation Pipeline (PGAAP) (version 2.4; http://www.ncbi.nlm.nih.gov/genome/annotation_prok/).

### Virulence factors in A. hydrophila

To summarize the known virulence genes of *A. hydrophila*, we read almost all of the articles discussing the pathogenicity of *A. hydrophila* that had been published between 1979 and 2014. The summarized virulence genes were then queried using BLASTp implemented in BLAST+ (version 2.2.29; ftp://ftp.ncbi.nlm.nih.gov/blast/executables/blast+/) against the six *A. hydrophila* genomes. A given virulence gene was deemed to be present in the genome once a gene shared more than 80% identity and covered at least 90% with it. To annotate the antibiotic genes and virulence genes of *A. hydrophila*, BLASTp was applied to align the amino acid sequences against ARDB and VFDB, respectively. Amino acid sequences with alignment lengths over 90% of their own lengths and with over 40% match identity were chosen, and the description of the best hit was assigned as the annotation of the predicted gene.

### Pathogenicity assays

The LD_50_s of *A. hydrophila* with zebrafish was performed according to our previous study^8^. For each *A. hydrophila* strain, eight groups of ten fish were intraperitoneally injected with 0.02 mL of serially tenfold-diluted bacterial suspensions containing 10^1^–10^8^ CFU. Members of the control group were injected intraperitoneally with 0.02 mL sterile PBS only. The zebrafish were observed until one week post-infection. For each determination, three separate experiments were performed. Survival data were analyzed according to a method devised by Reed and Muench^29^ for the calculation of LD_50_ values. ICR mice were used as a mammalian model to determine the virulence of *A. hydrophila*. Briefly, for each *A. hydrophila* strain, groups of ten female ICR mice were injected intraperitoneally with doses of 5.0 × 10^7^ CFU and 1.0 × 10^8^ CFU. Deaths were recorded daily for 14 days post-infection.

### Whole-genome comparisons

The phylogenetic network analysis was performed using 905 concatenated genes from the 16 *Aeromonas* genomes through the NeighborNet function in SplitsTree4 (version 4.13.1; http://www.splitstree.org/) on a set of 1000 bootstrap replicates to investigate the support for different branches in the network. To identify the core genome and the dispensable genome of *A. hydrophila*, preliminary analyses using the reciprocal BLASTp result file filtered with parameters of 90% minimum alignment length and 20–90% match identity were run. Then, a 90% minimum alignment length and an 80% minimum match identity were used to define orthology between two CDSs^30^. Finally, the core genome and dispensable genome among *Aeromonas* strains were calculated using in-house Perl scripts based on these criteria. It should be noted that the high-identity-sharing CDSs belonged to one genome and could be defined as single genes based on these criteria. To classify the functions encoded in the six *A. hydrophila* genomes, BLASTp was used to align the amino acid sequences against the COGs database. Amino acid sequences with alignment lengths of over 90% of their own length and over 40% match identity were chosen, and the description of the best hit was assigned as the annotation of the predicted genes. All annotated genes were then classified based on their COG classes.

### The identification of regions of genomic plasticity

RGPs were defined as regions that were longer than 10 kb or that contained at least four contiguous genes but that were absent at least in one genome analyzed^31^. The 27 RGPs used as the references were then queried using BLASTn implemented in BLAST + against the six *A. hydrophila* genomes, the alignment coverage was then summarized in Table 3 when the sequences shared more than 80% identity. In addition, the *A. hydrophila* genomes were submitted to the KEGG pathway database (updated Oct. 2, 2014; http://www.genome.jp/kegg/pathway.html) to analyze the metabolic pathways.

### MLST analysis and the distribution of putative virulence factors

MLST was performed as previously described^20^. Briefly, six housekeeping genes, *gyrB*, *groL*, *gltA, metG*, *ppsA* and *recA,* from each strain were submitted to the *Aeromonas* spp. MLST website to determine their identity against existing alleles. Each gene fragment was translated into a distinct allele, and each strain was classified into its sequence type by the combination of the alleles of the six housekeeping genes. New alleles that differed from the pre-existing alleles were assigned a new allele designation. For the phylogenetic analysis, six genes were aligned and concatenated using MEGA6 (version 6.0; http://www.megasoftware.net/). Then, the phylogenetic tree was constructed using the neighbor-joining method on a set of 1000 bootstrap replicates. Each of the virulence factors was detected by one to four primer pairs. All primers used in this study are listed in Table S9.

### Growth on myo-inositol, sialic acid and L-fucose

MEM medium (Yocon, CM0201) containing glutamine, but not phenol red, *myo*-inositol and glucose, was used as the carbon-restricted medium in this study. All chemicals were acquired from Sigma. Carbon sources such as *myo*-inositol, sialic acid, L-fucose and glucose were added to MEM to a final concentration of 0.1%. MEM containing glucose was used as a reference. To test the ability of *A. hydrophila* strains to utilize different carbon sources, overnight cultures grown in Luria broth (LB) were washed twice in MEM and inoculated into MEM containing different carbon sources at an OD_600_ of 0.01. Aliquots of these cultures (200 μL) were injected into 96-well plates, as were blanks containing only MEM. Each growth condition was replicated in 4 wells. Cultures were grown on a shaking incubator (80 rpm) at 28 °C, and the growth of each strain was detected by measuring the absorbance of the cultures at 570 nm using the Bio-Rad Model 680 microplate reader. Growth experiments for each strain were repeated on four separate occasions.

## Additional Information

**How to cite this article**: Pang, M. *et al.* Novel insights into the pathogenicity of epidemic *Aeromonas hydrophila* ST251 clones from comparative genomics. *Sci. Rep.* **5**, 9833; doi: 10.1038/srep09833 (2015).

## Supplementary Material

Supporting Information

Supporting Information

Supporting Information

Supporting Information

## Figures and Tables

**Table 1:** General features of *A. hydrophila* genomes.

**Strain**	**NJ-35**	**J-1**	**ML09-119**	**AL09-71**	**pc104A**	**ATCC 7966^T^**
Accession number	CP006870	CP006883	NC_021290	CP007566	CP007576	NC_008570
Status	Complete	Complete	Complete	Complete	Complete	Complete
Date of isolation	2010	1989	2009	2009	2010	Undetermined
Country of isolation	China	China	USA	USA	USA	USA
Source	Diseased carp	Diseased carp	Diseased catfish	Channel catfish	Pond soil	Fishy milk
Genome size (bp)	5,279,644	5,000,814	5,024,500	5,023,861	5,023,829	4,744,448
G + C content (%)	60.51	60.90	60.80	60.80	60.80	61.50
No. of genes	4,716	4,462	4,577	4492	4493	4,284
No. of CDS	4,526	4,268	4,434	4297	4300	4,119
No. of rRNAs	31	31	31	31	31	31
No. of tRNAs	102	110	112	111	111	128

**Table 2:** Putative virulence factors of *A. hydrophila* genomes.

	**Locus tag**						
**Virulence function**	**NJ-35**	**J-1**	**ML09-119**	**AL09-71**	**pc104A**	**ATCC 7966^T^**	**Reference**
Secretion system							
T2SS^a^	02520–02525	02535–02540	20110–20105	20950–20945	20980–20975	3786–3785	24
T3SS	NF	NF	NF	NF	NF	NF	21
T6SS	13500–13610	13090–13200	NF	NF	NF	1848–1826	22
Motility and adhesion							
Polar flagellum^b^	07230–07305	07255–07330	15330–15255	15855–15780	15890–15815	2847–2832	25
Lateral flagella	NF	NF	NF	NF	NF	NF	32
Flp type IV pilus	15530–15590	15125–15185	08135–08075	08365–08305	08370–08310	1462–1450	33
Msh type IV pilus	21860–21950	21205–21295	01980–01890	02115–02025	02115–02025	0399–0383	34
Tap type IV pilus^c^	02085–02095	02100–02110	20515–20500	21385–21375	21415–21405	3871–3868	33
Type IV pilus	21135–21170	20480–20515	02695–02660	02840–02805	02840–02805	0526–0518	13
O-antigen gene culsters	06980–07085	07000–07110	15485–15595	16000–16095	16035–16130	2908–2877	10
Toxin							
cytotoxic enterotoxin, AerA	21570	20915	02265	02405	02405	0438	35
heat-stable cytotonic enterotoxin, Ast	19690	19030	04100	04285	04285	0804	36
extracellular hemolysin, AHH1	15265	14860	08400	08630	08635	1512	37
hemolysin, HlyA	07430	07455	15145	15655	15690	2809	38
hemolysin III	03995	04025	18530	19245	19275	3493	13
thermostable hemolysin (TH)	05350	05370	17235	17890	17925	3217	39
RtxA	16300	15900	07370	07595	07600	1359	40
Enzyme							
AroA	09965	09595	13050	13505	13525	1979	41
DNA adenine methyltransferase, Dam	05505	05525	17080	17735	17770	3186	42
elastase, AhpB	19440	18780	04340	04535	04535	0851	43
enolase; Eno	19605	18945	04185	04370	04370	0821	44
extracellular protease, EprA1	08175	08205	14405	14915	14940	2713	45
glucose-inhibited division protein A, GidA	24435	23000	22245	23235	23270	4273	46
phospholipase A1, PLA	23360	22715	00550	00605	00605	0104	47
phospholipase C, PLC	20565	19910	03265	03410	03410	0635	47
exoribonuclease R, VacB	20220	19565	03605	03755	03755	0702	48
serine protease, SerA	08315	08330	14260	14760	14780	2687	49
ToxR-regulated lipoprotein, TagA	18525	17900	05230	05450	05455	0978	50
UDP N-acetylgalactosamine 4-epimerase, Gne	17585	17180	05955	06170	06170	1113	51
UDP-galactose-4-epimerase, GalE	00915	00920	21615	22575	22605	4103	51
UDP-glucose pyrophosphorylase, GalU	03965	03995	18560	19275	19305	3499	52
Quorum system							
AI-1 QS system	20975–20980	20320–20325	02850–02845	03000–02995	03000–02995	0557–0556	53
AI-2 QS system	20230	19575	03595	03745	03745	0700	54
QseBC QS system	05320–05325	05340–05345	17265–17260	17920–17915	17955–17950	3223–3222	55
Iron acquisition							
amonabactin synthesis and uptake	09855–09885	09485–09515	13155–13125	13615–13585	13635–13605	2479–2473	56
ferric uptake regulator, Fur	15170	14765	08495	08725	08730	1530	57
heme uptake	18575–18595	17950–17970	05180–05160	05400–05380	05405–05385	0968–0964	58
putative heme receptor	18555	17930	05200	05420	05425	0972	56
siderophore synthesis	05035–05040	05055–05060	17550–17545	18205–18200	18240–18235	3282–3281	13
Antibiotic resistance							
β-lactamase, AmpC	05765	05785	16865	17475	17510	3135	59
metallo-β-lactamase, CphA	20015	19360	03800	03960	03960	0740	60

^a,b,c,d^Only partial genes of these virulence factors were shown, and whole genes of them were shown in Table S1. NF represents “not found”. The beginning letters of locus tags (U876_, V469_, AHML_, V428_, V429_ and AHA_) are omitted.

**Table 3:** Regions of genomic plasticity identified in *A. hydrophila* genomes.
