# Supplementary material for: Novel insights into the pathogenicity of epidemic Aeromonas hydrophila ST251 clones from comparative genomics
Source: Sci Rep. 2015 May 27;5:9833. doi: 10.1038/srep09833 (PMC4444815; doi:10.1038/srep09833)
Supplement: Supporting Information [file srep09833-s4.pdf]

Novel insights into the pathogenicity of epidemic *Aeromonas hydrophila* ST251 clones from comparative genomics

Maoda Pang[1**], Jingwei Jiang[2**], Xing Xie[1], Yafeng Wu[1], Yuhao Dong[1], Amy H.Y. Kwok[2], Wei Zhang[1], Huochun Yao[1], Chengping Lu[1], Frederick C. Leung[2,3*] & Yongjie Liu[1*]

[1]College of Veterinary Medicine, Nanjing Agricultural University, Nanjing, 210095, China;

[2]Bioinformatics Center, Nanjing Agricultural University, Nanjing, 210095, China;

[3]School of Biological Sciences, University of Hong Kong, Hong Kong SAR, 999077, China

[**]M.D.P. and J.W.J. contributed equally to this article.

[*]Corresponding author: Yongjie Liu (liuyongjie@njau.edu.cn)

Frederick C. Leung (fcleung.njau@gmail.com)

Supplementary information

Table S1. Putative virulence factors of *A. hydrophila* genomes (supplementary).

Table S2. Antibiotic resistance genes of *A. hydrophila* genomes predicted using ARDB.

Table S3. Average nucleotide identity (ANI) values for *A. hydrophila* genomes and related *Aeromonas* species

Table S4. O-antigen gene clusters of *A. hydrophila.*

Table S5. Prophage regions identified by PHAST.

Table S6. Detail description of eight prophages.

Table S7. RGPs specific to epidemic *A. hydrophila.*

Table S8. *A. hydrophila* strains of ST251 clonal group.

Table S9. Primers used in this study.

Figure S1. Virulence of *A. hydrophila* strains assessed in zebrafish (a) and ICR mice (b). The virulence of *A. hydrophila* strains are depicted using LD_{50}s in zebrafish and survival of ICR mice, respectively. Statistical significance was analyzed by one-way analysis of variance (ANOVA), followed by Turkey's multiple comparison test.

Figure S2. Characterization of the prophage regions predicted in *A. hydrophila* NJ-35, J-1 and ML09-119. These prophage regions were predicted with PHAST.

Figure S3. Virulence of *A. hydrophila* strains assessed in zebrafish.

Dataset S1. Virulence genes of six *A. hydrophila* genomes.

Dataset S2. Genes categorized as COG G, L, P, R and S.

Dataset S3. 27 RGPs identified in *A. hydrophila* genomes.

Table S1. Putative virulence factors of *A. hydrophila* genomes (Supplementary)

Virulence function	Locus tag						Reference
	NJ-35	J-1	ML09-119	AL09-71	pc104A	ATCC 7966^T	
Secretion system							
T2SS (ExeAB)	02520-02525	02535-02540	20110-20105	20950-20945	20980-20975	3786-3785	1
T2SS (ExeN-C)	20860-20920	20205-20265	02965-02905	03115-03055	03115-03055	0579-0568	1
T2SS (TapD)	02085	02100	20515	21385	21415	3871	1
Motility and adhesion							
Polar flagellum	07230-07305	07255-07330	15330-15255	15855-15780	15890-15815	2847-2832	2
	07335-07345	07360-07370	15225-15215	15750-15740	15785-15775	2826-2824	
	14245-14285	13850-13880	09380-09350	09640-09610	09645-09615	1703-1698	
	16140-16275	15740-15875	07530-07395	07755-07620	07760-07625	1391-1364	
Tap type IV pilus	02085-02095	02100-02110	20515-20500	21385-21375	21415-21405	3871-3868	3
	03185-03190	03195-03200	19300-19295	20095-20090	20125-20120	3666-3665	
	05465-05485	05485-05505	17120-17100	17775-17755	17810-17790	3194-3190	
	03105	03115	19380	20175	20205	3683	
	08370	08385	14210	14705	14725	2681	
Iron acquisition							
amonabactin synthesis and uptake	09855-09885	09485-09515	13155-13125	13615-13585	13635-13605	2479-2473	4
	09890-09920	09520-09550	13120-13090	13580-13550	13600-13570	1964-1970	

The beginning letters of locus tags (U876_, V469_, AHML_, V428_, V429_ and AHA_) are omitted.

Table S2. Antibiotic resistance genes of *A. hydrophila* genomes predicted using ARDB

Type	Resistance	Strain NJ-35			Strain J-1			Strain ML09-119			Strain AL09-71			Strain pc104A			Strain ATCC 7966^T		
		Locus_tag	Identity (%)	Alignment length	Locus_tag	Identity (%)	Alignment length	Locus_tag	Identity (%)	Alignment length	Locus_tag	Identity (%)	Alignment length	Locus_tag	Identity (%)	Alignment length	Locus_tag	Identity (%)	Alignment length
acra	acriflavin	U876_06965	58.91	387	V469_06985	58.91	387	AHML_15605	62.39	335	V428_16105	62.39	335	V429_16140	62.39	335	AHA_2911	58.66	387
acrb	acriflavin	U876_06970	68.95	1050	V469_06990	68.95	1050	AHML_15600	69.74	1018	V428_16100	69.74	1018	V429_16135	69.74	1018	AHA_2910	68.86	1050
tolc	acriflavin	U876_02620	51.58	444	V469_02635	51.58	444	AHML_20005	51.84	434	V428_20850	51.58	444	V429_20880	51.58	444	AHA_3766	50.22	454
aac6if	amikacin	U876_08075	54.23	142	V469_08105	54.23	142	AHML_14505	54.23	142	V428_15015	54.23	142	V429_15040	54.23	142	AHA_2734	55.63	142
oprm	aminoglycoside	U876_06975	57.39	467	V469_06995	57.39	467	NF[a]	——	——	NF	——	——	NF	——	——	AHA_2909	57.39	467
bl3_cpha	carbapenem	U876_20015	98.81	253	V469_19360	98.81	253	AHML_03800	98.81	253	V428_03960	98.81	253	V429_03960	98.81	253	AHA_0740	100	253
bl1_ceps	cephalosporin	U876_05765	98.87	353	V469_05785	98.87	353	AHML_16865	98.87	353	V428_17475	98.87	353	V429_17510	98.87	353	AHA_3135	100	423
catb2	chloramphenicol	U876_00185	58.64	220	V469_00185	58.64	220	AHML_00165	58.64	220	V428_00185	58.64	220	V429_00185	58.64	220	AHA_0037	58.64	220
catb1	chloramphenicol	U876_03235	42.79	201	V469_03245	42.79	201	AHML_19250	42.79	201	V428_20045	42.79	201	V429_20075	42.79	201	AHA_3656	41.95	205
ceob	chloramphenicol	U876_06720	48.94	1042	V469_06740	48.94	1042	AHML_15985	48.82	1055	V428_16520	48.82	1055	V429_16555	48.82	1055	AHA_2960	48.82	1055
mdtl	chloramphenicol	U876_11755	41.44	362	V469_11335	41.21	364	AHML_11720	41.44	362	V428_12070	41.21	364	V429_12085	41.44	362	AHA_2243	41.71	362
norm	ciprofloxacin	U876_10620	57.79	443	V469_10205	57.79	443	AHML_12465	58.35	425	V428_12850	57.79	443	V429_12870	57.79	443	AHA_2114	57.73	440
bl2d_oxa9	cloxacillin	U876_24360	44.21	242	V469_22925	44.21	242	AHML_22170	44.21	242	V428_23160	44.21	242	V429_23195	44.21	242	AHA_4258	43.8	242
mdth	deoxycholate	U876_19430	58.58	367	V469_18770	58.58	367	AHML_04350	58.76	354	V428_04545	58.58	367	V429_04545	58.58	367	AHA_0853	58.58	367
qnra	fluoroquinolone	U876_22425	50.23	215	V469_21775	50.23	215	AHML_01435	50.23	215	V428_01545	50.23	215	V429_01545	50.23	215	AHA_0291	50.23	215
rosb	fosmidomycin	U876_06225	63.74	546	V469_06245	63.74	546	AHML_16445	63.74	546	V428_17015	63.74	546	V429_17050	63.74	546	AHA_3050	63.74	546
rosa	fosmidomycin	U876_00280	64.43	402	V469_00280	64.43	402	AHML_00325	64.43	402	V428_00355	64.43	402	V429_00355	64.43	402	AHA_0056	65.47	391
ksga	kasugamycin	U876_18990	68.05	266	V469_18370	68.05	266	AHML_04765	68.05	266	V428_04985	68.05	266	V429_04985	68.05	266	AHA_0942	68.05	266
macb	macrolide	U876_10155	44.24	217	V469_09780	44.24	217	AHML_12875	43.58	218	V428_13305	44.24	217	V429_13325	43.58	218	AHA_2006	44.7	217
pbp1a	penicillin	U876_05460	46.82	833	V469_05480	46.82	833	AHML_17125	46.82	833	V428_17780	46.82	833	V429_17815	46.82	833	AHA_3195	46.94	833
pbp1b	penicillin	U876_03815	40.11	698	V469_03845	40.11	698	AHML_18690	40.11	698	V428_19430	40.11	698	V429_19460	40.11	698	AHA_3530	40.11	698
tet34	tetracycline	U876_04315	63.58	151	V469_04335	63.58	151	AHML_18265	63.58	151	V428_18930	63.58	151	V429_18960	64.24	151	AHA_3424	63.58	151
dfra26	trimethoprim	U876_04550	42.07	164	V469_04570	42.07	164	AHML_18030	42.07	164	V428_18695	42.07	164	V429_18725	42.07	164	AHA_3377	42.07	164

[a] NF represents "not found".

Table S3. Average nucleotide identity (ANI) values for *A. hydrophila* genomes and related *Aeromonas* species

Strains	*A. hydrophila* NJ-35	*A. hydrophila* J-1	*A. hydrophila* ML09-119	*A. hydrophila* AL09-71	*A. hydrophila* pc104A	*A. hydrophila* ATCC7966^{T}	*A. veronii* AER39	*A. veronii* AER397	*A. veronii* AMC34	*A. veronii* AMC35	*A. Veronii* B565	*A. salmonicida* A449	*A. salmonicida* 01-B526	*A. media* WS	*A. caviae* Ae398	*A. dhakensis* AAK1
A. hydrophila NJ-35	---	**99.91**	**99.82**	**99.81**	**99.81**	**96.75**	85.47	85.47	85.45	85.31	85.68	86.5	86.44	86.24	86.41	92.92
A. hydrophila J-1	**99.98**	---	**99.86**	**99.86**	**99.86**	**96.79**	85.52	85.47	85.45	85.38	85.72	86.51	86.47	86.2	86.36	92.93
A. hydrophila ML09-119	**99.84**	**99.87**	---	**99.99**	**99.99**	**96.77**	85.51	85.5	85.45	85.51	85.74	86.42	86.4	86.19	86.27	92.79
A. hydrophila AL09-71	**99.84**	**99.86**	**99.99**	---	**100**	**96.77**	85.49	85.43	85.47	85.41	85.68	86.43	86.42	86.14	86.3	92.8
A. hydrophila pc104A	**99.84**	**99.85**	**99.99**	**100**	---	**96.77**	85.5	85.48	85.53	85.45	85.72	86.44	86.42	86.16	86.29	92.81
A. hydrophila ATCC7966^{T}	**96.76**	**96.74**	**96.77**	**96.77**	**96.77**	---	85.53	85.46	85.43	85.46	85.5	86.48	86.45	86.12	86.45	92.92
A. veronii AER39	85.55	85.63	85.6	85.6	85.6	85.56	---	96.42	93.74	96.46	96.43	84.22	84.26	84.1	83.78	85.17
A. veronii AER397	85.53	85.6	85.56	85.55	85.56	85.49	96.4	---	93.65	96.95	99.68	84.05	84.03	83.91	83.65	85.21
A. veronii AMC34	85.44	85.47	85.47	85.47	85.47	85.39	93.8	93.74	---	93.74	93.78	83.81	83.8	83.88	83.56	85.13
A. veronii AMC35	85.39	85.49	85.51	85.51	85.51	85.42	96.45	96.97	93.74	---	96.98	84.09	84.03	83.97	83.84	85.18
A. veronii B565	85.72	85.79	85.76	85.76	85.76	85.55	96.41	99.63	93.68	96.96	---	84.11	84.03	83.94	83.74	85.18
A. salmonicida A449	86.57	86.61	86.52	86.52	86.52	86.56	84.22	84.13	84.04	84.16	84.17	---	99.97	85.09	84.19	85.99
A. salmonicida 01-B526	86.21	86.25	86.17	86.17	86.17	86.22	84.02	83.86	83.69	83.86	83.89	98.98	---	85.11	83.92	86.03
A. media WS	86.22	86.27	86.27	86.26	86.27	86.25	84.01	83.93	83.83	83.91	83.94	85.08	85.1	---	88.07	86.09
A. caviae Ae398	86.33	86.4	86.35	86.35	86.35	86.48	83.61	83.63	83.56	83.74	83.63	83.96	83.95	88.07	---	86.54
A. dhakensis AAK1	92.99	93.02	92.92	92.91	92.91	92.96	85.2	85.18	85.04	85.23	85.2	85.98	86.02	85.99	86.54	---

ANIb was used in this study; Values in bold indicate strains that belong to *A. hydrophila* (ANI > 95)[5].

Table S4. O-antigen gene clusters of *A. hydrophila*

Strain	ORF ID	Locus	Start	End	Protein name	Putative function
NJ-35	up	U876_06975	1486984	1488393	OprM	Outer membrane protein OprM; multidrug transporter
	1	U876_06980	1489192	1490277	RmlB	dTDP-glucose 4,6-dehydratase
	2	U876_06985	1490277	1491164	RmlD	dTDP-4-dehydrorhamnose reductase
	3	U876_06990	1491277	1492155	RmlA	glucose-1-phosphate thymidylyltransferase
	4	U876_06995	1492214	1492771	RmlC	dTDP-4-dehydrorhamnose 3,5-epimerase
	5	U876_07005	1497191	1497439	Hyp	hypothetical protein
	6	U876_07010	1497868	1498053	Hyp	hypothetical protein
	7	U876_07015	1502039	1502668	GT	glycosyl transferase
	8	U876_07020	1502668	1503624	WbpK	NAD-dependent epimerase/dehydratase; UDP-glucose 4-epimerase
	9	U876_07025	1503624	1504649	GT	glycosyl transferase
	10	U876_07030	1504646	1506634	WbgZ	Putative polysaccharide biosynthesis protein; PII uridylyl-transferase
	11	U876_07035	1507118	1508185	WecA	UDP-phosphate alpha-N-acetylglucosaminyl 1-phosphate transferase
	12	U876_07040	1508867	1508289	Hyp	hypothetical protein
	13	U876_07045	1509436	1510716	WecC	UDP-glucose dehydrogenase; Vi polysaccharide biosynthesis protein VipA/TviB
	14	U876_07055	1516513	1517550	WbjB	UDP-glucose 4-epimerase
	15	U876_07060	1517553	1518434	RmlD	dTDP-4-dehydrorhamnose reductase
	16	U876_07065	1518422	1519555	WbjD	UDP-N-acetylglucosamine 2-epimerase
	17	U876_07070	1519552	1520760	GT	glycosyl transferase
	18	U876_07075	1521261	1522382	Wza	polysaccharide export protein
	19	U876_07080	1522992	1522693	Hyp	hypothetical protein
	20	U876_07085	1523106	1525280	Wzc	tyrosine protein kinase
	down	U876_07095	1527168	1529252	YjbH	membrane protein; Lipoprotein
J-1	up	V469_06995	1487989	1489398	OprM	Outer membrane protein OprM; multidrug transporter
	1	V469_07000	1490197	1491282	RmlB	dTDP-glucose 4,6-dehydratase
	2	V469_07005	1491282	1492169	RmlD	dTDP-4-dehydrorhamnose reductase
	3	V469_07010	1492282	1493160	RmlA	glucose-1-phosphate thymidylyltransferase
	4	V469_07015	1493219	1493776	RmlC	dTDP-4-dehydrorhamnose 3,5-epimerase
	5	V469_07025	1498196	1498444	Hyp	hypothetical protein
	6	V469_07030	1498873	1499058	Hyp	hypothetical protein
	7	V469_07035	1503038	1503667	GT	glycosyl transferase
	8	V469_07040	1503667	1504623	WbpK	NAD-dependent epimerase/dehydratase; UDP-glucose 4-epimerase
	9	V469_07045	1504623	1505648	GT	glycosyl transferase
	10	V469_07050	1505645	1507633	WbgZ	Putative polysaccharide biosynthesis protein; PII uridylyl-transferase
	11	V469_07055	1508117	1509184	WecA	UDP-phosphate alpha-N-acetylglucosaminyl 1-phosphate transferase
	12	V469_07060	1509866	1509288	Hyp	hypothetical protein
	13	V469_07065	1510435	1511715	WecC	UDP-glucose dehydrogenase; Vi polysaccharide biosynthesis protein VipA/TviB
	14	V469_07080	1517514	1518551	WbjB	UDP-glucose 4-epimerase
	15	V469_07085	1518554	1519435	RmlD	dTDP-4-dehydrorhamnose reductase

	16	V469_07090	1519423	1520556	WbjD	UDP-N-acetylglucosamine 2-epimerase
	17	V469_07095	1520553	1521761	GT	glycosyl transferase
	18	V469_07100	1522262	1523383	Wza	polysaccharide export protein
	19	V469_07105	1523993	1523694	Hyp	hypothetical protein
	20	V469_07110	1524107	1526281	Wzc	tyrosine protein kinase
	down	V469_07120	1528169	1530253	YjbH	membrane protein; Lipoprotein
ML09-119	up	AHML_15480	3502937	3502266	YmcC	putative lipoprotein
	1	AHML_15485	3504107	3503379	WzzA	lipopolysaccharide biosynthesis protein
	2	AHML_15490	3507049	3504425	Wza	polysaccharide export protein
	3	AHML_15495	3508277	3507195	WzzB	O-antigen chain length determinant protein
	4	AHML_15500	3508871	3508332	RmlC	dTDP-4-dehydrorhamnose 3,5-epimeraseprotein; RfbC2
	5	AHML_15505	3509826	3508885	RmlA	glucose-1-phosphate thymidylyltransferase
	6	AHML_15510	3510608	3509826	RmlD	dTDP-4-keto-L-rhamnose reductase
	7	AHML_15515	3511758	3510697	WecA	undecaprenyl-phosphate alpha-N-acetylglucosaminyl 1-phosphatetransferase
	8	AHML_15520	3513233	3511809	ManB	phosphomannomutase
	9	AHML_15525	3513985	3513230	GT	glycosyl transferase
	10	AHML_15530	3515388	3513982	ManC	mannose-1-phosphate guanylyltransferase
	11	AHML_15535	3515845	3515393	Gmm	GDP-mannose mannosyl hydrolase; NUDIX hydrolase
	12	AHML_15540	3516839	3515859	Fcl	GDP-L-fucose synthetase; Colanic acidbiosynthesis protein wcaG
	13	AHML_15545	3517481	3516843	Gmd	GDP-mannose 4,6-dehydratase
	14	AHML_15550	3518935	3517970	GT	group 1 glycosyl transferase
	15	AHML_15555	3520251	3519202	GT	group 1 glycosyl transferase
	16	AHML_15560	3520739	3520251	GT	group 1 glycosyl transferase
	17	AHML_15565	3521920	3521429	AT	acetyltransferase (isoleucine patch superfamily)-like protein
	18	AHML_15570	3525959	3524709	WzxB	WzxB protein; polysaccharide biosynthesis protein
	19	AHML_15575	3527059	3525956	FdtB	glutamine--scyllo-inositol transaminase; Aminotransferase
	20	AHML_15580	3527522	3527061	FdtC	WxcM-like protein;dTDP-d-fucp3n acetyltransferaseisomerase
	21	AHML_15585	3527922	3527500	FdtA	WxcM-like protein; dtdp-6-deoxy-3,4-keto-hexulose isomerase
	22	AHML_15590	3528832	3527933	RmlA	glucose-1-phosphate thymidylyltransferase
	23	AHML_15595	3529884	3528796	RmlB	dTDP-glucose 4,6 dehydratase
	down	AHML_15600	3533648	3530499	AcrB	Multidrug transporter; AcrB protein
AL09-71[a]	up	V428_15995	3501794	3501048		hypothetical protein
	1	V428_16000	3503830	3502904	WzzA	lipopolysaccharide biosynthesis protein
	2	V428_16005	3506574	3503950	Wza	polysaccharide export protein
	3	V428_16010	3507802	3506720	WzzB	O-antigen chain length determinant protein
	4	V428_16015	3507947	3508441	RmlC	dTDP-4-dehydrorhamnose 3,5-epimeraseprotein; RfbC2
	5	V428_16020	3509351	3508410	RmlA	glucose-1-phosphate thymidylyltransferase
	6	V428_16025	3510133	3509351	RmlD	dTDP-4-keto-L-rhamnose reductase
	7	V428_16030	3511283	3510222	WecA	undecaprenyl-phosphate alpha-N-acetylglucosaminyl 1-phosphatetransferase
	8	V428_16035	3512127	3511924	ManB	phosphomannomutase

	9	V428_16045	3514913	3513507	ManC	mannose-1-phosphate guanylyltransferase
	10	V428_16055	3516364	3515384	Fcl	GDP-L-fucose synthetase; Colanic acidbiosynthesis protein wcaG
	11	V428_16060	3517474	3516368	Gmd	GDP-mannose 4,6-dehydratase
	12	V428_16065	3520957	3519776	GT	group 1 glycosyl transferase
	13	V428_16075	3525484	3524234	WzxB	WzxB protein; polysaccharide biosynthesis protein
	14	V428_16080	3526584	3525481	FdtB	glutamine--scyllo-inositol transaminase; Aminotransferase
	15	V428_16090	3527692	3527976	RmlA	glucose-1-phosphate thymidylyltransferase
	16	V428_16095	3529409	3528321	RmlB	dTDP-glucose 4,6 dehydratase
	down	V428_16100	3533173	3530024	AcrB	Multidrug transporter; AcrB protein
pc104A[b]	up	V429_16030	3501766	3501020		hypothetical protein
	1	V429_16035	3503802	3502876	WzzA	lipopolysaccharide biosynthesis protein
	2	V429_16040	3506546	3503922	Wza	polysaccharide export protein
	3	V429_16045	3507774	3506692	WzzB	O-antigen chain length determinant protein
	4	V429_16050	3507919	3508413	RmlC	dTDP-4-dehydrorhamnose 3,5-epimeraseprotein; RfbC2
	5	V429_16055	3509323	3508382	RmlA	glucose-1-phosphate thymidylyltransferase
	6	V429_16060	3510105	3509323	RmlD	dTDP-4-keto-L-rhamnose reductase
	7	V429_16065	3511255	3510194	WecA	undecaprenyl-phosphate alpha-N-acetylglucosaminyl 1-phosphatetransferase
	8	V429_16070	3512099	3511896	ManB	phosphomannomutase
	9	V429_16080	3514885	3513479	ManC	mannose-1-phosphate guanylyltransferase
	10	V429_16090	3516336	3515356	Fcl	GDP-L-fucose synthetase; Colanic acidbiosynthesis protein wcaG
	11	V429_16095	3517446	3516340	Gmd	GDP-mannose 4,6-dehydratase
	12	V429_16100	3520929	3519748	GT	group 1 glycosyl transferase
	13	V429_16110	3525456	3524206	WzxB	WzxB protein; polysaccharide biosynthesis protein
	14	V429_16115	3526556	3525453	FdtB	glutamine--scyllo-inositol transaminase; Aminotransferase
	15	V429_16125	3527664	3527948	RmlA	glucose-1-phosphate thymidylyltransferase
	16	V429_16130	3529381	3528293	RmlB	dTDP-glucose 4,6 dehydratase
	down	V429_16135	3533145	3529996	AcrB	Multidrug transporter; AcrB protein
ATCC 7966[T]	up	AHA_2876	3230749	3230060	YmcC	lipoprotein
	1	AHA_2877	3233370	3231196	Wzc	tyrosine-protein kinase
	2	AHA_2878	3233861	3233433	Wzb	low molecular weight protein-tyrosine-phosphatase ptp
	3	AHA_2879	3235213	3234098	Wza	polysaccharide export protein
	4	AHA_2880	3236715	3235549	Ugd	UDP-glucose 6-dehydrogenase
	5	AHA_2881	3237741	3236728	WcvA	nucleotide sugar epimerase; UDP-glucuronate 4-epimerase
	6	AHA_2882	3238838	3237765	Hyp	hypothetical protein
	7	AHA_2883	3239671	3238865	GT	glycosyl transferase family protein
	8	AHA_2884	3240494	3239673	GT	glycosyl transferase family protein
	9	AHA_2885	3241592	3240498	GT	glycoside hydrolase family protein
	10	AHA_2886	3242869	3241655	Wzx	polysaccharide biosynthesis protein
	11	AHA_2887	3243986	3242871	Ald	alanine dehydrogenase; NAD(P) transhydrogenase subunit alpha
	12	AHA_2888	3245001	3243979	Hyp	hypothetical protein

13	AHA_2889	3246139	3245060	WecA	undecaprenyl-phosphate alpha-N-acetylglucosaminyl 1-phosphatetransferase
14	AHA_2890	3246596	3247573	Hyp	hypothetical protein
15	AHA_2891	3249649	3247664	WbgZ	nucleoside-diphosphate sugar epimerase; PII uridylyl-transferase
16	AHA_2892	3250671	3249646	GT	Glycosyl transferase, group 4 family protein; glycoside hydrolase family protein
17	AHA_2893	3251675	3250671	WbpK	NAD-dependent dehydratase; UDP-glucose 4-epimerase
18	AHA_2894	3252765	3251641	GT	group 1 glycosyl transferase
19	AHA_2895	3253231	3252743	Gmm	GDP-mannose mannosyl hydrolase
20	AHA_2896	3253863	3253246	AT	acetyltransferase
21	AHA_2897	3255508	3253868	GT	Glycosyl transferase；glycoside hydrolase family protein
22	AHA_2898	3257784	3255499	GT	glycosytransferase
23	AHA_2899	3258539	3257781	Wzt	ABC tranporter system ATP binding protein; O-antigen export system ATP-binding protein
24	AHA_2900	3259321	3258536	Wzm	ABC transporter permease
25	AHA_2901	3260474	3259386	Per	perosamine synthetase
26	AHA_2902	3261598	3260477	Gmd	GDP-mannose 4,6-dehydratase
27	AHA_2903	3262977	3261613	ManB	phosphomannomutase
28	AHA_2904	3264405	3263008	ManC	mannose-1-phosphate guanylyltransferase
29	AHA_2905	3264971	3264408	RmlC	dTDP-4-dehydrorhamnose 3,5-epimerase
30	AHA_2906	3265912	3265028	RmlA	glucose-1-phosphate thymidylyltransferase
31	AHA_2907	3266912	3266025	RmlD	dTDP-4-dehydrorhamnose reductase
32	AHA_2908	3267997	3266912	RmlB	dTDP-glucose-4,6-dehydratase
down	AHA_2909	3270174	3268759	OprM	Outer membrane protein OprM; multidrug transporter

[a, b]The O-antigen gene clusters of strains ML09-119, Al09-71 and pc104A were identical, however, because of the three genomes were annotated in different time and the genes in the O-antigen gene clusters were highly variable, the amounts of CDSs were different.

Table S5. Prophage regions identified by PHAST

Prophage ID	Strain as reference	Completeness (score)	Specific keyword	Showup of att site	Coordinate	Region length (kbp)
Prophage-1	NJ-35	incomplete (50)	transposase, tail, capsid	No	1704008-1711889	7.8
Prophage-2	NJ-35	incomplete (20)	integrase	Yes	1917892-1944140	26.2
Prophage-3	NJ-35	intact (110)	lysin, tail, terminase, portal	No	1935562-1981545	45.9
Prophage-4	NJ-35	questionable (80)	integrase, transposase, tail	Yes	2049761-2067289	17.5
Prophage-5	NJ-35	questionable (70)	plate, terminase, portal, tail	No	2481289-2510319	29
Prophage-6	NJ-35	intact (100)	tail, head, protease, virion, portal, transposase, integrase	Yes	3414289-3444499	30.2
Prophage-7	J-1	incomplete (40)	tail, transposase, integrase	Yes	3978435-4006936	28.5
Prophage-8	ML09-119	intact (150)	integrase, portal,terminase, capsid, head, tail	Yes	4399658-4437862	38.2

Table S6. Detail descriptions of eight prophages

Prophage	ID	CDS_POSITION	BLAST_HIT	E-VALUE
Prophage-1 (strain NJ-35)	1	1704008..1704601	PROPHAGE_Escher_CFT073: insertion element IS2 transposase InsD; PP_01580; phage(gi26249446)	5e-86
	2	complement(1704630..1704758)	AraC family transcriptional regulator [Aeromonas hydrophila ML09-119] gi|507523754|ref|YP_008045368.1|; PP_01581	5e-09
	3	1705081..1705614	alkylhydroperoxidase AhpD domain-containing protein [Aeromonas hydrophila ML09-119] gi|507522441|ref|YP_008044055.1|; PP_01582	7e-94
	4	complement(1705611..1705754)	hypothetical; PP_01583	0.0
	5	1705761..1706063	MC21 protein [Aeromonas hydrophila ML09-119] gi|507522440|ref|YP_008044054.1|; PP_01584	2e-51
	6	complement(1706888..1707667)	PHAGE_Salmon_RE_2010_NC_019488: gene D protein; PP_01585; phage(gi418489726)	7e-53
	7	complement(1707738..1708694)	PHAGE_Phaeoc_virus_NC_021312: FkbM family methyltransferase; PP_01586; phage(gi508181807)	2e-14
	8	complement(1708964..1709152)	PHAGE_Salmon_RE_2010_NC_019488: tail completion protein; PP_01587; phage(gi418489707)	8e-05
	9	1709381..1709878	PHAGE_Salmon_RE_2010_NC_019488: capsid packaging protein; PP_01588; phage(gi418489696)	3e-35
	10	1709875..1710012	PHAGE_Salmon_RE_2010_NC_019488: capsid packaging protein; PP_01589; phage(gi418489696)	4e-06
	11	1710711..1711889	PHAGE_Escher_TL_2011b_NC_019445: hypothetical protein; PP_01590; phage(gi418487678)	2e-08
Prophage-2 (strain NJ-35)	1	1917892..1917903	attL TTTGTTCATGGG	0.0
	2	complement(1917965..1919218)	PHAGE_Salmon_Fels_1_NC_010391: putative bacteriophage integrase; PP_01777; phage(gi169257156)	7e-63
	3	complement(1919218..1919424)	PHAGE_Bordet_BPP_1_NC_005357: Bbp49; PP_01778; phage(gi41179409)	7e-06
	4	complement(1919388..1919798)	PHAGE_Lactob_phiadh_NC_000896: hypothetical protein phiadhp07; PP_01779; phage(gi9633007)	2e-06
	5	1919958..1920149	hypothetical; PP_01780	0.0
	6	complement(1920194..1920901)	PHAGE_Entero_HK629_NC_019711: hypothetical protein; PP_01781; phage(gi428782044)	1e-06
	7	complement(1920977..1921204)	hypothetical; PP_01782	0.0
	8	complement(1921276..1921494)	hypothetical; PP_01783	0.0
	9	complement(1921458..1922585)	PHAGE_Salmon_vB_SosS_Oslo_NC_018279: hypothetical protein; PP_01784; phage(gi399528795)	4e-11
	10	complement(1922582..1923202)	PHAGE_Entero_HK97_NC_002167: Gp37; PP_01785; phage(gi9634184)	1e-07
	11	complement(1923199..1923543)	hypothetical; PP_01786	0.0
	12	complement(1923540..1924478)	PHAGE_Entero_P1_NC_005856: HrdC; PP_01787; phage(gi46401690)	1e-15
	13	complement(1924499..1924621)	hypothetical; PP_01788	0.0
	14	complement(1924737..1925339)	PHAGE_Aeromo_vB_AsaM_56_NC_019527: putative Dam methylase; PP_01790; phage(gi422937495)	2e-63
	15	complement(1925339..1925479)	hypothetical; PP_01789	0.0
	16	complement(1925476..1926696)	PHAGE_Vibrio_12A4_NC_021068: hypothetical protein; PP_01791; phage(gi481019127)	8e-23
	17	complement(1926750..1927838)	PHAGE_Pseudo_vB_PaeP_Tr60_Ab31_NC_023575: Hypothetical protein; PP_01792; phage(gi589286911)	3e-65
	18	complement(1927911..1928885)	PHAGE_Salmon_SPN1S_NC_016761: putative RecT family protein; PP_01793; phage(gi374531229)	2e-56
	19	complement(1928882..1929724)	PHAGE_Salmon_SPN1S_NC_016761: putative exonuclease VIII/RecE-like protein; PP_01794; phage(gi374531231)	5e-86
	20	complement(1929721..1930113)	hypothetical; PP_01795	0.0
	21	complement(1930239..1930604)	hypothetical; PP_01796	0.0
	22	complement(1930601..1930849)	PHAGE_Aeromo_vB_AsaM_56_NC_019527: hypothetical protein; PP_01797; phage(gi422937499)	2e-08
	23	complement(1930846..1931043)	hypothetical protein AHA_2021 [Aeromonas hydrophila subsp. hydrophila ATCC 7966] gi|117619931|ref|YP_856551.1|; PP_01798	9e-22
	24	complement(1931118..1931804)	PHAGE_Bacter_APSE_2_NC_011551: conserved hypothetical protein G; PP_01799; phage(gi212499739)	1e-16
	25	1944140..1944151	attR TTTGTTCATGGG	0.0
	1	1935562..1936716	PHAGE_Mycoba_Bruin_NC_022988: DNA methylase; PP_01806; phage(gi563399694)	4e-72
	2	1936779..1937696	PHAGE_Entero_mEp237_NC_019704: DNA replication protein O; PP_01807; phage(gi435439307)	8e-49
	3	1937696..1938367	PHAGE_Pseudo_vB_PaeP_Tr60_Ab31_NC_023575: Putative replication protein P; PP_01808; phage(gi589286927)	1e-24

	4	1938354..1938656	hypothetical protein AHML_04890 [Aeromonas hydrophila ML09-119] gi|507520495|ref|YP_008042109.1|; PP_01809	3e-11
	5	1938796..1939026	hypothetical; PP_01810	0.0
	6	1939023..1939553	PHAGE_Escher_TL_2011c_NC_019442: phage regulatory protein, Rha family; PP_01811; phage(gi418487059)	1e-22
	7	1939550..1939990	PHAGE_Entero_ST104_NC_005841: NinB; PP_01812; phage(gi46358678)	8e-35
	8	1939987..1940304	PHAGE_Burkho_BcepMigl_NC_019917: hypothetical protein; PP_01813; phage(gi431809874)	5e-15
Prophage 3 (strain NJ-35)	9	1940301..1940435	hypothetical; PP_01814	0.0
	10	1940498..1940854	PHAGE_Vibrio_12A4_NC_021068: hypothetical protein; PP_01815; phage(gi481019085)	2e-28
	11	1940851..1941552	hypothetical protein AHA_2042 [Aeromonas hydrophila subsp. hydrophila ATCC 7966] gi|117617719|ref|YP_856572.1|; PP_01816	2e-113
	12	complement(1941827..1941955)	hypothetical; PP_01817	0.0
	13	complement(1942868..1944148)	PHAGE_Cronob_ENT39118_NC_019934: DNA polymerase; PP_01818; phage(gi431811050)	6e-131
	14	complement(1944145..1944567)	PHAGE_Cronob_ENT39118_NC_019934: protein umuD; PP_01819; phage(gi431811072)	6e-26
	15	complement(1944578..1944691)	hypothetical; PP_01820	0.0
	16	1944691..1944885	hypothetical; PP_01821	0.0
	17	1945484..1945696	PHAGE_Entero_vB_EcoP_ACG_C91_NC_019403: putative holin; PP_01822; phage(gi414087620)	7e-06
	18	1945903..1946184	PHAGE_Acinet_AP22_NC_017984: putative endolysin/autolysin; PP_01823; phage(gi388570824)	3e-11
	19	1946434..1946682	PHAGE_Salmon_E1_NC_010495: tail protein; PP_01824; phage(gi170676286)	4e-06
	20	1946760..1946939	hypothetical; PP_01825	0.0
	21	1946940..1947809	PHAGE_Escher_TL_2011c_NC_019442: putative terminase small subunit; PP_01826; phage(gi418487072)	2e-45
	22	1947843..1948202	hypothetical; PP_01827	0.0
	23	1948261..1949958	PHAGE_Entero_Min27_NC_010237: putative large subunit terminase; PP_01828; phage(gi170783661)	0.0
	24	1949958..1952084	PHAGE_Escher_TL_2011c_NC_019442: putative portal protein; PP_01829; phage(gi418487074)	0.0
	25	1952206..1952541	PHAGE_Burkho_BcepMigl_NC_019917: hypothetical protein; PP_01830; phage(gi431809906)	1e-18
	26	1952673..1953602	PHAGE_Escher_P13374_NC_018846: hypothetical protein; PP_01831; phage(gi410491654)	9e-40
	27	1953674..1954891	PHAGE_Escher_TL_2011c_NC_019442: hypothetical protein; PP_01832; phage(gi418487102)	3e-132
	28	1954957..1955346	PHAGE_Escher_TL_2011c_NC_019442: hypothetical protein; PP_01833; phage(gi418487103)	1e-07
	29	1955413..1955853	PHAGE_Escher_TL_2011c_NC_019442: hypothetical protein; PP_01834; phage(gi418487104)	8e-27
	30	1955856..1956437	PHAGE_Escher_TL_2011c_NC_019442: hypothetical protein; PP_01835; phage(gi418487105)	6e-16
	31	1956447..1957124	PHAGE_Escher_TL_2011c_NC_019442: hypothetical protein; PP_01836; phage(gi418487106)	6e-41
	32	1957137..1958864	PHAGE_Escher_bV_EcoS_AKFV33_NC_017969: putative tail fiber protein; PP_01837; phage(gi388570497)	4e-38
	33	1958861..1959184	hypothetical; PP_01838	0.0
	34	1959226..1960884	PHAGE_Stx2_converting_86_NC_008464: hypothetical protein Stx2-86_gp25; PP_01839; phage(gi116222017)	2e-131
	35	1960938..1964726	PHAGE_Salmon_SSU5_JQ965645: putative phage tail protein; PP_01840; phage(gi390013855)	3e-37
	36	1964865..1965092	PHAGE_Escher_TL_2011c_NC_019442: hypothetical protein; PP_01841; phage(gi418487115)	1e-13
	37	1965102..1965734	PHAGE_Stx2_converting_I_NC_003525: hypothetical protein Stx2Ip042; PP_01842; phage(gi20065838)	4e-46
	38	1965745..1966125	PHAGE_Escher_TL_2011c_NC_019442: hypothetical protein; PP_01843; phage(gi418487117)	3e-24
	39	1966125..1966385	PHAGE_Escher_TL_2011c_NC_019442: hypothetical protein; PP_01844; phage(gi418487118)	4e-17
	40	1966395..1967795	PHAGE_Escher_TL_2011c_NC_019442: hypothetical protein; PP_01845; phage(gi418487119)	8e-22
	41	1967927..1974238	PHAGE_Thalas_BA3_NC_009990: hypothetical protein BA3_0002; PP_01846; phage(gi160700596)	2e-57
	42	1974317..1974520	hypothetical; PP_01847	0.0
	43	complement(1974521..1974862)	PHAGE_Escher_TL_2011c_NC_019442: hypothetical protein; PP_01849; phage(gi418487100)	1e-12
	44	complement(1974871..1974996)	hypothetical; PP_01848	0.0
	45	complement(1975205..1975324)	hypothetical; PP_01850	0.0

	46	complement(1975399..1975767)	PHAGE_Salmon_vB_SemP_Emek_NC_018275: transcriptional repressor protein; PP_01851; phage(gi399498811)	1e-07
	47	1976116..1976937	PHAGE_Vibrio_ICP1_NC_015157: putative DNA-binding protein Roi; PP_01852; phage(gi325171078)	9e-43
	48	1976934..1977065	hypothetical; PP_01853	0.0
	49	complement(1977441..1977734)	hypothetical; PP_01854	0.0
	50	1978172..1978291	hypothetical; PP_01855	0.0
	51	1978574..1979458	PHAGE_Vibrio_VH7D_NC_023568: hypothetical protein; PP_01856; phage(gi589286286)	1e-14
	52	complement(1979533..1981545)	PHAGE_Acinet_IME_AB3_NC_023590: putative tail tape measure protein; PP_01857; phage(gi589892167)	3e-06
Prophage-4 (strain NJ-35)	1	2049761..2049772	attL AAAGGGATACGA	0.0
	2	2050112..2051419	PROPHAGE_Xantho_306: integrase; PP_01923; phage(gi21242250)	9e-15
	3	complement(2051630..2051752)	hypothetical; PP_01924	0.0
	4	2052131..2053327	PHAGE_Bordet_BMP_1_NC_005808: integrase; PP_01925; phage(gi45569541)	2e-21
	5	2053465..2054427	hypothetical protein AHML_13505 [Aeromonas hydrophila ML09-119] gi|507522204|ref|YP_008043818.1|; PP_01926	2e-180
	6	complement(2054901..2055062)	hypothetical; PP_01927	0.0
	7	complement(2055076..2055192)	hypothetical; PP_01928	0.0
	8	2055392..2056453	PHAGE_Mycoba_Muddy_NC_022054: RecA; PP_01929; phage(gi530545338)	1e-09
	9	2056591..2056806	hypothetical; PP_01930	0.0
	10	complement(2057216..2058661)	transferrin-binding protein A [Aeromonas hydrophila ML09-119] gi|507522201|ref|YP_008043815.1|; PP_01931	2e-93
	11	complement(2058633..2059337)	transferrin-binding protein A [Edwardsiella tarda FL6-60] gi|387867510|ref|YP_005698979.1|; PP_01932	3e-53
	12	2059390..2059536	hypothetical; PP_01933	0.0
	13	2059586..2059852	PROPHAGE_Xantho_306: ISxcd1 transposase; PP_01934; phage(gi21243158)	1e-37
	14	2059873..2060001	PROPHAGE_Xantho_306: ISxcd1 transposase; PP_01935; phage(gi21242257)	5e-07
	15	2060273..2060575	PROPHAGE_Ralsto_GMI1000: ISRSO10-transposase ORFA protein; PP_01936; phage(gi17546153)	2e-39
	16	2061261..2061869	PROPHAGE_Escher_CFT073: transposase insF; PP_01937; phage(gi26249410)	1e-82
	17	complement(2062494..2063096)	PHAGE_Thermo_THSA_485A_NC_018264: transcriptional regulator, XRE family; PP_01938; phage(gi397912660)	2e-07
	18	2063140..2063835	hypothetical protein AHML_13455 [Aeromonas hydrophila ML09-119] gi|507522194|ref|YP_008043808.1|; PP_01939	3e-130
	19	complement(2063971..2065083)	alanine racemase [Aeromonas hydrophila ML09-119] gi|507522193|ref|YP_008043807.1|; PP_01940	0.0
	20	complement(2065559..2067190)	PHAGE_Lactob_LF1_NC_019486: tail fiber; PP_01941; phage(gi418489400)	4e-08
	21	2067289..2067300	attR AAAGGGATACGA	0.0
Prophage-5 (strain NJ-35)	1	2481289..2481606	PHAGE_Serrat_Eta_NC_021563: holin; PP_02329; phage(gi514361091)	1e-11
	2	2481606..2482085	PHAGE_Pectob_PM1_NC_023865: TIGR02594 family protein; PP_02330; phage(gi602993987)	2e-56
	3	2482141..2482365	PHAGE_Vibrio_X29_NC_024369: hypothetical protein SBVcX29_0035; PP_02331; phage(gi658311202)	6e-07
	4	2482445..2482597	hypothetical; PP_02332	0.0
	5	2482651..2482830	hypothetical; PP_02333	0.0
	6	2482831..2483721	PHAGE_Escher_TL_2011c_NC_019442: putative terminase small subunit; PP_02334; phage(gi418487072)	5e-46
	7	2484027..2484419	hypothetical; PP_02335	0.0
	8	2484475..2486172	PHAGE_Entero_Min27_NC_010237: putative large subunit terminase; PP_02336; phage(gi170783661)	0.0
	9	2486172..2488298	PHAGE_Escher_TL_2011c_NC_019442: putative portal protein; PP_02337; phage(gi418487074)	0.0
	10	2488594..2489532	PHAGE_Escher_P13374_NC_018846: hypothetical protein; PP_02338; phage(gi410491654)	1e-39
	11	2489604..2490821	PHAGE_Escher_TL_2011c_NC_019442: hypothetical protein; PP_02339; phage(gi418487102)	6e-133
	12	2490885..2491274	PHAGE_Escher_TL_2011c_NC_019442: hypothetical protein; PP_02340; phage(gi418487103)	7e-08
	13	2491336..2491776	PHAGE_Escher_TL_2011c_NC_019442: hypothetical protein; PP_02341; phage(gi418487104)	1e-27
	14	2491779..2492360	PHAGE_Escher_TL_2011c_NC_019442: hypothetical protein; PP_02342; phage(gi418487105)	2e-17

	15	2492370..2493047	PHAGE_Escher_TL_2011c_NC_019442: hypothetical protein; PP_02343; phage(gi418487106)	5e-41
	16	2493060..2494697	PHAGE_Escher_bV_EcoS_AKFV33_NC_017969: putative tail fiber protein; PP_02344; phage(gi388570497)	6e-24
	17	2494694..2495017	hypothetical; PP_02345	0.0
	18	2495059..2496717	PHAGE_Stx2_converting_86_NC_008464: hypothetical protein Stx2-86_gp25; PP_02346; phage(gi116222017)	8e-133
	19	2496717..2500607	PHAGE_Stx2_converting_86_NC_008464: putative tail tip fiber protein; PP_02347; phage(gi116222018)	7e-35
	20	2500604..2500963	PHAGE_Escher_TL_2011c_NC_019442: hypothetical protein; PP_02348; phage(gi418487115)	7e-19
	21	2500973..2501605	PHAGE_Stx2_converting_I_NC_003525: hypothetical protein Stx2Ip042; PP_02349; phage(gi20065838)	3e-43
	22	2501616..2501996	PHAGE_Escher_TL_2011c_NC_019442: hypothetical protein; PP_02350; phage(gi418487117)	4e-22
	23	2502053..2502235	PHAGE_Escher_TL_2011c_NC_019442: hypothetical protein; PP_02351; phage(gi418487118)	2e-06
	24	2502245..2503645	PHAGE_Escher_TL_2011c_NC_019442: hypothetical protein; PP_02352; phage(gi418487119)	6e-22
	25	2503777..2510319	PHAGE_Thalas_BA3_NC_009990: hypothetical protein BA3_0002; PP_02353; phage(gi160700596)	5e-58
Prophage-6 (strain NJ-35)	1	complement(3414289..3417132)	PHAGE_Stenot_S1_NC_011589: putative tape measure protein; PP_03189; phage(gi213163916)	1e-28
	2	3415047..3415060	attL CGGCGGTGGTCTTG	0.0
	3	3417227..3417859	hypothetical protein Geob_3129 [Geobacter daltonii FRC-32] gi|222056211|ref|YP_002538573.1|; PP_03190	2e-10
	4	3418057..3418341	hypothetical protein AHML_07985 [Aeromonas hydrophila ML09-119] gi|507521114|ref|YP_008042728.1|; PP_03191	5e-51
	5	3418527..3419294	hypothetical protein B565_1789 [Aeromonas veronii B565] gi|330829489|ref|YP_004392441.1|; PP_03192	2e-55
	6	complement(3419353..3420105)	PHAGE_Stenot_S1_NC_011589: putative tail protein a; PP_03193; phage(gi213163914)	7e-16
	7	complement(3420102..3420548)	PHAGE_Pseudo_JBD24_NC_020203: hypothetical protein; PP_03194; phage(gi448245089)	7e-06
	8	complement(3420545..3420958)	PHAGE_Haemop_SuMu_NC_019455: Mu-like prophage protein gp36; PP_03195; phage(gi418489087)	6e-18
	9	complement(3420960..3421409)	PHAGE_Pseudo_B3_NC_006548: hypothetical protein B3ORF39; PP_03196; phage(gi56692608)	9e-06
	10	complement(3421513..3422421)	PHAGE_Escher_D108_NC_013594: major head protein; PP_03197; phage(gi281199678)	5e-101
	11	complement(3422466..3423614)	PHAGE_Escher_D108_NC_013594: protease; PP_03198; phage(gi281199676)	3e-82
	12	complement(3423837..3424283)	PHAGE_Vibrio_12B12_NC_021070: phage virion morphogenesis protein; PP_03199; phage(gi481019160)	2e-06
	13	complement(3424283..3424483)	hypothetical protein AHML_07945 [Aeromonas hydrophila ML09-119] gi|507521106|ref|YP_008042720.1|; PP_03200	2e-29
	14	complement(3424503..3425972)	PHAGE_Vibrio_12B12_NC_021070: F protein; PP_03201; phage(gi481019167)	2e-38
	15	complement(3425972..3427561)	PHAGE_Escher_D108_NC_013594: portal protein; PP_03202; phage(gi281199672)	3e-124
	16	complement(3427561..3429114)	PHAGE_Vibrio_12B12_NC_021070: portal protein; PP_03203; phage(gi481019169)	4e-162
	17	complement(3429114..3429689)	PHAGE_Vibrio_12B12_NC_021070: hypothetical protein; PP_03204; phage(gi481019171)	2e-34
	18	complement(3429692..3429988)	PHAGE_Vibrio_12B12_NC_021070: hypothetical protein; PP_03205; phage(gi481019172)	1e-22
	19	complement(3429985..3430299)	PHAGE_Vibrio_12B12_NC_021070: hypothetical protein; PP_03206; phage(gi481019173)	7e-14
	20	complement(3430280..3430507)	PHAGE_Mannhe_vB_MhM_1152AP_NC_021778: putative zinc-finger containing protein; PP_03207; phage(gi525972265)	4e-06
	21	complement(3430529..3430750)	hypothetical protein B565_1773 [Aeromonas veronii B565] gi|330829473|ref|YP_004392425.1|; PP_03208	1e-32
	22	complement(3430743..3431045)	hypothetical protein AHML_07900 [Aeromonas hydrophila ML09-119] gi|507521097|ref|YP_008042711.1|; PP_03209	1e-51
	23	complement(3431042..3431617)	PHAGE_Vibrio_VHML_NC_004456: ORF19; PP_03210; phage(gi27311185)	1e-15
	24	complement(3432248..3432832)	hypothetical protein AHML_07890 [Aeromonas hydrophila ML09-119] gi|507521095|ref|YP_008042709.1|; PP_03211	1e-107
	25	complement(3433061..3433402)	hypothetical Protein AHML_07885 [Aeromonas hydrophila ML09-119] gi|507521094|ref|YP_008042708.1|; PP_03212	5e-62
	26	complement(3433585..3434022)	PHAGE_Vibrio_12B12_NC_021070: hypothetical protein; PP_03213; phage(gi481019179)	1e-15
	27	complement(3434012..3434638)	PHAGE_Vibrio_12B12_NC_021070: hypothetical protein; PP_03214; phage(gi481019180)	1e-25
	28	complement(3434635..3434805)	PHAGE_Vibrio_12B12_NC_021070: hypothetical protein; PP_03215; phage(gi481019185)	3e-05
	29	complement(3434792..3435028)	hypothetical protein AHML_07870 [Aeromonas hydrophila ML09-119] gi|507521091|ref|YP_008042705.1|; PP_03216	3e-35
	30	complement(3435146..3435340)	hypothetical protein AHML_07865 [Aeromonas hydrophila ML09-119] gi|507521090|ref|YP_008042704.1|; PP_03217	6e-31
	31	complement(3435355..3435975)	PHAGE_Vibrio_12B12_NC_021070: hypothetical protein; PP_03218; phage(gi481019189)	4e-61

	32	complement(3435986..3436165)	hypothetical protein AHML_07855 [Aeromonas hydrophila ML09-119] gi|507521088|ref|YP_008042702.1|; PP_03219	6e-27
	33	complement(3436177..3436419)	hypothetical protein AHML_07850 [Aeromonas hydrophila ML09-119] gi|507521087|ref|YP_008042701.1|; PP_03220	1e-39
	34	complement(3436484..3437035)	PHAGE_Rhodov_RS1_NC_020866: hypothetical protein; PP_03221; phage(gi472342885)	1e-11
	35	complement(3437700..3438422)	PHAGE_Rhodov_RS1_NC_020866: transposase; PP_03222; phage(gi472342883)	3e-23
	36	3438287..3438300	attR CGGCGGTGGTCTTG	0.0
	37	complement(3438466..3440679)	PHAGE_Rhodov_RS1_NC_020866: integrase; PP_03223; phage(gi472342882)	3e-52
	38	3441115..3441906	PHAGE_Pseudo_JD024_NC_024330: repressor; PP_03224; phage(gi658307175)	2e-36
	39	3442205..3444499	PHAGE_Bacill_BCJA1c_NC_006557: DEAD box family helicase; PP_03225; phage(gi56694884)	6e-23
Prophage-7 (strain J-1)	1	3978435..3978447	attL TGCCAAGCGGCGG	0.0
	2	complement(3985512..3986573)	PHAGE_Aeromo_phiO18P_NC_009542: putative N6-methyltransferase; PP_03714; phage(gi148727180)	5e-133
	3	complement(3987156..3989027)	hypothetical protein VP2143 [Vibrio parahaemolyticus RIMD 2210633] gi|28898917|ref|NP_798522.1|; PP_03715	4e-88
	4	complement(3989037..3991577)	PHAGE_Staphy_aureus_P68_NC_004679: minor tail protein; PP_03716; phage(gi29565757)	4e-06
	5	complement(3992138..3992338)	PROPHAGE_Escher_CFT073: transposase insF; PP_03717; phage(gi26250329)	1e-11
	6	complement(3992507..3993127)	PHAGE_Entero_P22_NC_002371: transcription antitermination protein; PP_03718; phage(gi51236742)	6e-25
	7	complement(3993127..3993417)	PHAGE_Salmon_SPN1S_NC_016761: hypothetical protein; PP_03719; phage(gi374531238)	3e-25
	8	complement(3993501..3994490)	PHAGE_Entero_mEp460_NC_019716: hypothetical protein; PP_03720; phage(gi428782365)	3e-24
	9	complement(3994487..3994750)	hypothetical protein AHML_04915 [Aeromonas hydrophila ML09-119] gi|507520500|ref|YP_008042114.1|; PP_03721	3e-39
	10	complement(3995879..3996361)	hypothetical protein AHML_04905 [Aeromonas hydrophila ML09-119] gi|507520498|ref|YP_008042112.1|; PP_03722	1e-85
	11	complement(3996576..3997274)	PHAGE_Entero_YYZ_2008_NC_011356: DNA-binding protein Roi; PP_03723; phage(gi209427760)	1e-35
	12	complement(3997291..3997545)	hypothetical protein AHML_04890 [Aeromonas hydrophila ML09-119] gi|507520495|ref|YP_008042109.1|; PP_03724	6e-42
	13	complement(3997545..3997952)	hypothetical protein AHML_04885 [Aeromonas hydrophila ML09-119] gi|507520494|ref|YP_008042108.1|; PP_03725	7e-70
	14	complement(3998014..3999135)	PHAGE_Entero_mEp390_NC_019721: hypothetical protein; PP_03726; phage(gi428782706)	4e-25
	15	complement(3999128..3999253)	hypothetical; PP_03727	0.0
	16	complement(4000609..4000785)	Lambda phage phage regulatory protein CII [Aeromonas hydrophila ML09-119] gi|507520491|ref|YP_008042105.1|; PP_03728	8e-24
	17	4001941..4002105	transcriptional repressor pyocin R2 PP [Aeromonas hydrophila ML09-119] gi|507520489|ref|YP_008042103.1|; PP_03729	3e-24
	18	4003046..4003267	hypothetical protein AHML_04850 [Aeromonas hydrophila ML09-119] gi|507520487|ref|YP_008042101.1|; PP_03730	2e-34
	19	4003305..4004291	PHAGE_Salmon_ST64B_NC_004313: putative DNA methyltransferase; PP_03731; phage(gi23505488)	1e-53
	20	4004701..4004895	hypothetical protein AHML_04835 [Aeromonas hydrophila ML09-119] gi|507520484|ref|YP_008042098.1|; PP_03732	7e-30
	21	4004977..4005234	hypothetical protein AHML_04830 [Aeromonas hydrophila ML09-119] gi|507520483|ref|YP_008042097.1|; PP_03733	2e-42
	22	4005234..4005374	PHAGE_Vibrio_X29_NC_024369: single-stranded DNA-binding protein; PP_03734; phage(gi658311176)	2e-09
	23	4005301..4005313	attR TGCCAAGCGGCGG	0.0
	24	4005629..4006936	PHAGE_Stx2_converting_II_NC_004914: integrase; PP_03735; phage(gi302393112)	1e-80
	1	complement(4399658..4401010)	PHAGE_Acanth_mimivirus_NC_014649: putative RNA methyltransferase; PP_04105; phage(gi311977789)	2e-25
	2	complement(4401324..4402760)	ammonium transporter [Aeromonas hydrophila ML09-119] gi|507523389|ref|YP_008045003.1|; PP_04106	0.0
	3	4403218..4403267	attL CGGGGTCGGACTCGAACCGACACGGTTATTCACCGGCGGATTTTGAATCC	0.0
	4	complement(4403337..4404257)	PHAGE_Aeromo_phiO18P_NC_009542: putative integrase; PP_04107; phage(gi148727179)	5e-162
	5	complement(4404373..4404717)	PHAGE_Aeromo_phiO18P_NC_009542: hypothetical protein phiO18_3; PP_04109; phage(gi148727132)	2e-48
	6	4404982..4405149	hypothetical; PP_04108	0.0
	7	complement(4405234..4405947)	PHAGE_Aeromo_phiO18P_NC_009542: putative cI repressor; PP_04110; phage(gi148727133)	5e-102
	8	4406329..4406838	PHAGE_Aeromo_phiO18P_NC_009542: putative cII protein; PP_04111; phage(gi148727135)	3e-78
	9	4406849..4407307	PHAGE_Aeromo_phiO18P_NC_009542: hypothetical protein phio18_7; PP_04112; phage(gi148727136)	5e-59
	10	4407565..4407828	PHAGE_Salmon_SP_004_NC_021774: hypothetical protein; PP_04113; phage(gi526003642)	3e-08

	11	4407831..4408004	PHAGE_Aeromo_phiO18P_NC_009542: hypothetical protein phiO18_8; PP_04114; phage(gi148727137)	7e-21
	12	4408001..4408192	PHAGE_Aeromo_phiO18P_NC_009542: hypothetical protein phiO18_9; PP_04115; phage(gi148727138)	4e-22
Prophage-8	13	4408189..4408398	PHAGE_Aeromo_phiO18P_NC_009542: hypothetical protein phiO18_10; PP_04116; phage(gi148727139)	3e-30
(strain ML09-119)	14	4408533..4408811	hypothetical protein AHML_19505 [Aeromonas hydrophila ML09-119] gi|507523399|ref|YP_008045013.1|; PP_04117	3e-45
	15	4408808..4409344	PHAGE_Vibrio_8_NC_022747: putative DNA methyltransferase; PP_04118; phage(gi557307486)	3e-67
	16	4409595..4411928	PHAGE_Aeromo_phiO18P_NC_009542: putative replication protein; PP_04119; phage(gi148727142)	0.0
	17	4412199..4412696	PHAGE_Aeromo_phiO18P_NC_009542: hypothetical protein phiO18_14; PP_04120; phage(gi148727143)	2e-80
	18	4413178..4413396	PHAGE_Aeromo_phiO18P_NC_009542: hypothetical protein phiO18_16; PP_04121; phage(gi148727145)	5e-25
	19	4413393..4413521	hypothetical; PP_04122	0.0
	20	4413514..4413846	PHAGE_Pectob_ZF40_NC_019522: hypothetical protein; PP_04123; phage(gi422936662)	2e-10
	21	4414083..4414907	hypothetical protein [Vibrio furnissii NCTC 11218] gi|375131177|ref|YP_004993277.1|; PP_04124	5e-09
	22	complement(4415231..4415584)	hypothetical protein AHML_19540 [Aeromonas hydrophila ML09-119] gi|507523406|ref|YP_008045020.1|; PP_04125	8e-50
	23	4415748..4415918	hypothetical protein AHML_19545 [Aeromonas hydrophila ML09-119] gi|507523407|ref|YP_008045021.1|; PP_04126	3e-24
	24	complement(4415961..4416212)	PHAGE_Aeromo_phiO18P_NC_009542: hypothetical protein phiO18_19; PP_04127; phage(gi148727148)	9e-38
	25	complement(4416278..4417294)	PHAGE_Aeromo_phiO18P_NC_009542: putative portal protein; PP_04128; phage(gi148727149)	0.0
	26	complement(4417291..4419111)	PHAGE_Aeromo_phiO18P_NC_009542: putative terminase large subunit; PP_04129; phage(gi148727151)	0.0
	27	4419383..4420144	PHAGE_Aeromo_phiO18P_NC_009542: putative capsid scaffolding protein; PP_04130; phage(gi148727152)	3e-116
	28	4420154..4421203	PHAGE_Aeromo_phiO18P_NC_009542: putative major capsid protein; PP_04131; phage(gi148727153)	2e-173
	29	4421249..4421935	PHAGE_Aeromo_phiO18P_NC_009542: putative terminase small subunit; PP_04132; phage(gi148727154)	5e-116
	30	4422106..4422567	PHAGE_Aeromo_phiO18P_NC_009542: putative head completion protein; PP_04133; phage(gi148727155)	2e-73
	31	4422576..4423088	PHAGE_Aeromo_phiO18P_NC_009542: hypothetical protein phiO18_27; PP_04134; phage(gi148727156)	3e-86
	32	4423085..4423771	PHAGE_Aeromo_phiO18P_NC_009542: putative tail completion protein; PP_04135; phage(gi148727157)	4e-113
	33	4423776..4424900	PHAGE_Aeromo_phiO18P_NC_009542: putative tail sheath protein; PP_04136; phage(gi148727158)	3e-176
	34	4424904..4425359	PHAGE_Aeromo_phiO18P_NC_009542: putative tail tube protein; PP_04137; phage(gi148727159)	2e-72
	35	4425363..4425572	PHAGE_Aeromo_phiO18P_NC_009542: putative zinc finger protein; PP_04138; phage(gi148727160)	3e-18
	36	4425594..4425920	PHAGE_Aeromo_phiO18P_NC_009542: putative holin; PP_04139; phage(gi148727161)	2e-28
	37	4425907..4426368	PHAGE_Aeromo_phiO18P_NC_009542: putative muramidase; PP_04140; phage(gi148727162)	4e-74
	38	4426365..4426808	PHAGE_Pseudo_PA1/KOR/2010_NC_023700: hypothetical protein; PP_04141; phage(gi593775464)	9e-05
	39	4426783..4426932	PHAGE_Aeromo_phiO18P_NC_009542: hypothetical protein phiO18_35; PP_04142; phage(gi148727163)	3e-18
	40	4426932..4427195	PHAGE_Aeromo_phiO18P_NC_009542: hypothetical protein phiO18_36; PP_04143; phage(gi148727164)	7e-35
	41	4427387..4429156	PHAGE_Aeromo_phiO18P_NC_009542: putative tail tape measure protein; PP_04144; phage(gi148727166)	0.0
	42	4429156..4429479	PHAGE_Aeromo_phiO18P_NC_009542: hypothetical protein phiO18_39; PP_04145; phage(gi148727167)	2e-48
	43	4429476..4430663	PHAGE_Aeromo_phiO18P_NC_009542: hypothetical protein phiO18_40; PP_04146; phage(gi148727168)	2e-180
	44	4430656..4431336	PHAGE_Aeromo_phiO18P_NC_009542: hypothetical protein phiO18_41; PP_04147; phage(gi148727169)	6e-87
	45	4431336..4434239	PHAGE_Aeromo_phiO18P_NC_009542: putative tail fiber protein; PP_04148; phage(gi158518662)	2e-96
	46	4434353..4434949	PHAGE_Aeromo_phiO18P_NC_009542: hypothetical protein phiO18_44; PP_04149; phage(gi148727173)	3e-49
	47	4434961..4435509	PHAGE_Aeromo_phiO18P_NC_009542: hypothetical protein phiO18_45; PP_04150; phage(gi148727174)	4e-64
	48	4435506..4437116	PHAGE_Aeromo_phiO18P_NC_009542: hypothetical protein phiO18_46; PP_04151; phage(gi148727175)	0.0
	49	4437862..4437911	attR CGGGGTCGGACTCGAACCGACACGGTTATTCACCGGCGGATTTTGAATCC	0.0

Table S7. RGPs specific to epidemic *A. hydrophila*

Specific RGPs	Strain	Locus_tag	Gene name	Start	End	Size of gene	Predicted function
myo-Inositol utilization gene clusters (RGP3)	NJ-35	U876_07795	iolA	1687409	1685898	1512	methylmalonate-semialdehyde dehydrogenase
		U876_07800	iolR	1687773	1688627	855	RpiR family transcriptional regulator
		U876_07805	iolD	1688776	1690710	1935	3D-(3,5/4)-trihydroxycyclohexane-1,2-dione hydrolase
		U876_07810	hyp	1691050	1691484	435	hypothetical protein
		U876_07820	iolG_2	1692506	1693492	987	myo-inositol 2-dehydrogenase
		U876_07825	rbsB	1693524	1694453	930	ABC-type ribose/inositol transport system substrate-binding protein
		U876_07830	rbsA	1694502	1696049	1548	ABC-type ribose/inositol transport system ATP-binding protein
		U876_07835	rbsC	1696061	1697089	1029	ABC-type ribose/inositol transport system permease protein
		U876_07840	iolG_1	1697098	1698231	1134	myo-inositol 2-dehydrogenase
		U876_07845	iolC	1698271	1700148	1878	5-dehydro-2-deoxygluconokinase
		U876_07850	iolE	1700160	1701050	891	inosose dehydratase
		U876_07855	iolB	1701102	1701923	822	5-deoxyglucuronate isomerase
	J-1	V469_07825	iolA	1688404	1686893	1512	methylmalonate-semialdehyde dehydrogenase
		V469_07830	iolR	1688768	1689622	855	RpiR family transcriptional regulator
		V469_07835	iolD	1689771	1691705	1935	3D-(3,5/4)-trihydroxycyclohexane-1,2-dione hydrolase
		V469_07840	hyp	1692045	1692479	435	hypothetical protein
		V469_07850	iolG_2	1693501	1694487	987	myo-inositol 2-dehydrogenase
		V469_07855	rbsB	1694519	1695448	930	ABC-type ribose/inositol transport system substrate-binding protein
		V469_07860	rbsA	1695497	1697044	1548	ABC-type ribose/inositol transport system ATP-binding protein
		V469_07865	rbsC	1697056	1698084	1029	ABC-type ribose/inositol transport system permease protein
		V469_07870	iolG_1	1698093	1699226	1134	myo-inositol 2-dehydrogenase
		V469_07875	iolC	1699266	1701143	1878	5-dehydro-2-deoxygluconokinase
		V469_07880	iolE	1701155	1702045	891	inosose dehydratase
		V469_07885	iolB	1702097	1702918	822	5-deoxyglucuronate isomerase
	ML09-119	AHML_14790	iolA	3341231	3342742	1512	methylmalonate-semialdehyde dehydrogenase
		AHML_14785	iolR	3340867	3340013	855	RpiR family transcriptional regulator
		AHML_14780	iolD	3339864	3337930	1935	3D-(3,5/4)-trihydroxycyclohexane-1,2-dione hydrolase
		AHML_14775	integrase	3337162	3338424	1263	integrase catalytic subunit
		AHML_14770	transposase	3336678	3336932	255	transposase IS3/IS911 family protein
		AHML_14765	iolG_2	3336133	3335147	987	myo-inositol 2-dehydrogenase
		AHML_14760	rbsB	3335115	3334186	930	ABC-type ribose/inositol transport system substrate-binding protein
		AHML_14755	rbsA	3334137	3332590	1548	ABC-type ribose/inositol transport system ATP-binding protein
		AHML_14750	rbsC	3332578	3331550	1029	ABC-type ribose/inositol transport system permease protein
		AHML_14745	iolG_1	3331541	3330408	1134	myo-inositol 2-dehydrogenase
		AHML_14740	iolC	3330395	3328491	1905	5-dehydro-2-deoxygluconokinase
		AHML_14735	iolE	3328479	3327589	891	inosose dehydratase
		AHML_14730	iolB	3327537	3326716	822	5-deoxyglucuronate isomerase
		V428_15290	iolA	3340756	3342267	1512	methylmalonate-semialdehyde dehydrogenase
		V428_15285	iolR	3340392	3339538	855	RpiR family transcriptional regulator
		V428_15280	iolD	3339389	3337455	1935	3D-(3,5/4)-trihydroxycyclohexane-1,2-dione hydrolase

		V428_15275	hyp	3337115	3336681	435	hypothetical protein
	AL09-71	V428_15270	iolG_2	3335658	3334672	987	myo-inositol 2-dehydrogenase
		V428_15265	rbsB	3334640	3333711	930	ABC-type ribose/inositol transport system substrate-binding protein
		V428_15260	rbsA	3333662	3332115	1548	ABC-type ribose/inositol transport system ATP-binding protein
		V428_15255	rbsC	3332103	3331075	1029	ABC-type ribose/inositol transport system permease protein
		V428_15250	iolG_1	3331066	3329933	1134	myo-inositol 2-dehydrogenase
		V428_15245	iolC	3329893	3328016	1878	5-dehydro-2-deoxygluconokinase
		V428_15240	iolE	3328004	3327114	891	inosose dehydratase
		V428_15235	iolB	3327062	3326241	822	5-deoxyglucuronate isomerase
		V429_15315	iolA	3340729	3342240	1512	methylmalonate-semialdehyde dehydrogenase
		V429_15310	iolR	3340365	3339511	855	RpiR family transcriptional regulator
		V429_15305	iolD	3339362	3337428	1935	3D-(3,5/4)-trihydroxycyclohexane-1,2-dione hydrolase
		V429_15300	hyp	3337088	3336654	435	hypothetical protein
		V429_15295	iolG_2	3335631	3334645	987	myo-inositol 2-dehydrogenase
	pc104A	V429_15290	rbsB	3334613	3333684	930	ABC-type ribose/inositol transport system substrate-binding protein
		V429_15285	rbsA	3333635	3332088	1548	ABC-type ribose/inositol transport system ATP-binding protein
		V429_15280	rbsC	3332076	3331048	1029	ABC-type ribose/inositol transport system permease protein
		V429_15275	iolG_1	3331039	3329906	1134	myo-inositol 2-dehydrogenase
		V429_15270	iolC	3329866	3327989	1878	5-dehydro-2-deoxygluconokinase
		V429_15265	iolE	3327977	3327087	891	inosose dehydratase
		V429_15260	iolB	3327035	3326214	822	5-deoxyglucuronate isomerase
		U876_16515	nanT	3601446	3599965	1482	N-acetylneuraminate transporter
		U876_16520	nanK	3602396	3601479	918	N-acetylmannosamine kinase
		U876_16525	nanE	3603100	3602393	708	N-acetylmannosamine-6-phosphate 2-epimerase
		U876_16530	nanR	3604323	3603451	873	rpiR-family Transcriptional regulator
		U876_16535	nanA	3604604	3605503	900	N-acetylneuraminate lyase
		U876_16540	katG	3606135	3608342	2208	peroxidase
		U876_16545	fucR	3609175	3609912	738	L-fucose operon activator
		U876_16550	fucP	3610054	3611274	1221	L-fucose permease
		U876_16555	fucI	3611284	3613062	1779	L-fucose isomerase
		U876_16560	fucK	3613129	3614577	1449	L-fuculose kinase
		U876_16565	fucA	3614574	3615416	843	L-fuculose-phosphate aldolase
	NJ-35	U876_16570	fucU	3615426	3615854	429	L-fucose mutarotase
		U876_16575	fucO	3615962	3617110	1149	Lactaldehyde reductase
		U876_16580	oppD	3618716	3617316	1401	ABC-type dipeptide/oligopeptide/nickel transport systems,ATP-binding protein
		U876_16585	oppC	3619509	3618709	801	ABC-type dipeptide/oligopeptide/nickel transport systems, permease components
		U876_16590	oppB	3620449	3619499	951	ABC-type dipeptide/oligopeptide/nickel transport systems, permease components
		U876_16595	oppA	3621963	3620449	1515	ABC-type dipeptide/oligopeptide/nickel transport systems, periplasmic component
		V469_16110	nanT	3530779	3529298	1482	N-acetylneuraminate transporter
		V469_16115	nanK	3531729	3530812	918	N-acetylmannosamine kinase
	J-1	V469_16120	nanE	3532433	3531726	708	N-acetylmannosamine-6-phosphate 2-epimerase
Sialic and L-fucose utilization		V469_16125	nanR	3533656	3532784	873	rpiR-family Transcriptional regulator

gene clusters (RGP13)		V469_16130	nanA	3533937	3534836	900	N-acetylneuraminate lyase
		V469_16135	katG	3535468	3537675	2208	peroxidase
		V469_16140	fucR	3538508	3539245	738	L-fucose operon activator
		V469_16145	fucP	3539387	3540607	1221	L-fucose permease
		V469_16150	fucI	3540617	3542395	1779	L-fucose isomerase
		V469_16155	fucK	3542462	3543910	1449	L-fuculose kinase
		V469_16160	fucA	3543907	3544749	843	L-fuculose-phosphate aldolase
		V469_16165	fucU	3544759	3545187	429	L-fucose mutarotase
		V469_16170	fucO	3545295	3546443	1149	Lactaldehyde reductase
		V469_16175	oppD	3548049	3546649	1401	ABC-type dipeptide/oligopeptide/nickel transport systems,ATP-binding protein
		V469_16180	oppC	3548842	3548042	801	ABC-type dipeptide/oligopeptide/nickel transport systems, permease components
		V469_16185	oppB	3549782	3548832	951	ABC-type dipeptide/oligopeptide/nickel transport systems, permease components
		V469_16190	oppA	3551296	3549782	1515	ABC-type dipeptide/oligopeptide/nickel transport systems, periplasmic component
	ML09-119	AHML_07165	nanT	1577869	1579350	1482	N-acetylneuraminate transporter
		AHML_07160	nanK	1576919	1577836	918	N-acetylmannosamine kinase
		AHML_07155	nanE	1576296	1576922	627	N-acetylmannosamine-6-phosphate 2-epimerase
		AHML_07150	nanR	1574992	1575864	873	rpiR-family Transcriptional regulator
		AHML_07145	nanA	1574711	1573812	900	N-acetylneuraminate lyase
		AHML_07140	katG	1573180	1570973	2208	peroxidase
		AHML_07135	fucR	1569403	1569948	546	L-fucose operon activator
		AHML_07130	fucP	1569249	1568041	1209	L-fucose permease
		AHML_07125	fucI	1568031	1566253	1779	L-fucose isomerase
		AHML_07120	fucK	1566186	1564738	1449	L-fuculose kinase
		AHML_07115	fucA	1564741	1563899	843	L-fuculose-phosphate aldolase
		AHML_07110	fucU	1563889	1563461	429	L-fucose mutarotase
		AHML_07105	fucO	1563353	1562205	1149	Lactaldehyde reductase
		AHML_07100	oppD	1560599	1561999	1401	ABC-type dipeptide/oligopeptide/nickel transport systems,ATP-binding protein
		AHML_07095	oppC	1559806	1560606	801	ABC-type dipeptide/oligopeptide/nickel transport systems, permease components
		AHML_07090	oppB	1558866	1559816	951	ABC-type dipeptide/oligopeptide/nickel transport systems, permease components
		AHML_07085	oppA	1557352	1558866	1515	ABC-type dipeptide/oligopeptide/nickel transport systems, periplasmic component
	AL09-71	V428_07385	nanT	1577540	1579021	1482	N-acetylneuraminate transporter
		V428_07380	nanK	1576590	1577507	918	N-acetylmannosamine kinase
		V428_07375	nanE	1575886	1576593	708	N-acetylmannosamine-6-phosphate 2-epimerase
		V428_07370	nanR	1574663	1575535	873	rpiR-family Transcriptional regulator
		V428_07365	nanA	1574382	1573483	900	N-acetylneuraminate lyase
		V428_07360	katG	1572851	1570644	2208	peroxidase
		V428_07355	fucR	1569811	1569074	738	L-fucose operon activator
		V428_07350	fucP	1568932	1567712	1221	L-fucose permease
		V428_07345	fucI	1567702	1565924	1779	L-fucose isomerase
		V428_07340	fucK	1565857	1564409	1449	L-fuculose kinase
		V428_07335	fucA	1564412	1563570	843	L-fuculose-phosphate aldolase
		V428_07330	fucU	1563560	1563132	429	L-fucose mutarotase

		V428_07325	fucO	1563024	1561876	1149	Lactaldehyde reductase
		V428_07320	oppD	1560270	1561670	1401	ABC-type dipeptide/oligopeptide/nickel transport systems,ATP-binding protein
		V428_07315	oppC	1559477	1560277	801	ABC-type dipeptide/oligopeptide/nickel transport systems, permease components
		V428_07310	oppB	1558537	1559487	951	ABC-type dipeptide/oligopeptide/nickel transport systems, permease components
		V428_07305	oppA	1557023	1558537	1515	ABC-type dipeptide/oligopeptide/nickel transport systems, periplasmic component
	pc104A	V429_07390	nanT	1577539	1579020	1482	N-acetylneuraminate transporter
		V429_07385	nanK	1576589	1577506	918	N-acetylmannosamine kinase
		V429_07380	nanE	1575885	1576592	708	N-acetylmannosamine-6-phosphate 2-epimerase
		V429_07375	nanR	1574662	1575534	873	rpiR-family Transcriptional regulator
		V429_07370	nanA	1574381	1573482	900	N-acetylneuraminate lyase
		V429_07365	katG	1572850	1570643	2208	peroxidase
		V429_07360	fucR	1569810	1569073	738	L-fucose operon activator
		V429_07355	fucP	1568931	1567711	1221	L-fucose permease
		V429_07350	fucI	1567701	1565923	1779	L-fucose isomerase
		V429_07345	fucK	1565856	1564408	1449	L-fuculose kinase
		V429_07340	fucA	1564411	1563569	843	L-fuculose-phosphate aldolase
		V429_07335	fucU	1563559	1563131	429	L-fucose mutarotase
		V429_07330	fucO	1563023	1561875	1149	Lactaldehyde reductase
		V429_07325	oppD	1560269	1561669	1401	ABC-type dipeptide/oligopeptide/nickel transport systems,ATP-binding protein
		V429_07320	oppC	1559476	1560276	801	ABC-type dipeptide/oligopeptide/nickel transport systems, permease components
		V429_07315	oppB	1558536	1559486	951	ABC-type dipeptide/oligopeptide/nickel transport systems, permease components
		V429_07310	oppA	1557022	1558536	1515	ABC-type dipeptide/oligopeptide/nickel transport systems, periplasmic component
ABC-type transport systems (RGP15)	NJ-35	U876_18070	fecA_1	3938553	3940625	2073	TonB-dependent receptor
		U876_18075	pdtP	3940628	3941698	1071	Methyltransferase type 12
		U876_18080	ssuA	3941695	3942723	1029	ABC-type nitrate/sulfonate/bicarbonate transport system. substrate-binding protein
		U876_18085	ssuC	3942710	3943462	753	ABC-type nitrate/sulfonate/bicarbonate ABC transport system, permease protein
		U876_18090	ssuB	3943459	3944133	675	ABC-type nitrate/sulfonate/bicarbonate transport system, ATP-binding protein
		U876_18095	norR	3944346	3944819	474	Anaerobic nitric oxide reductase transcription regulator
		U876_18100	fecA_2	3944929	3946968	2040	TonB-dependent receptor
		U876_18105	fnr	3947739	3947023	717	Fumarate and nitrate reduction regulatory protein
		U876_18110	fecB	3947836	3948915	1080	ABC-type Fe3+-siderophore transport system, substrate-binding protein
		U876_18115	fecC	3948915	3949934	1020	ABC-type Fe3+-siderophore transport system, permease component
		U876_18120	fecE	3949931	3950671	741	ABC-type Fe3+-siderophore transport system, ATP-binding protein
	J-1	V469_17445	fecA_1	3827010	3829082	2073	TonB-dependent receptor
		V469_17450	pdtP	3829085	3830155	1071	Methyltransferase type 12
		V469_17455	ssuA	3830152	3831180	1029	ABC-type nitrate/sulfonate/bicarbonate transport system. substrate-binding protein
		V469_17460	ssuC	3831167	3831919	753	ABC-type nitrate/sulfonate/bicarbonate ABC transport system, permease protein
		V469_17465	ssuB	3831916	3832590	675	ABC-type nitrate/sulfonate/bicarbonate transport system, ATP-binding protein
		V469_17470	norR	3832804	3833277	474	Anaerobic nitric oxide reductase transcription regulator
		V469_17475	fecA_2	3833387	3835426	2040	TonB-dependent receptor
		V469_17480	fnr	3836197	3835481	717	Fumarate and nitrate reduction regulatory protein
		V469_17485	fecB	3836294	3837373	1080	ABC-type Fe3+-siderophore transport system, substrate-binding protein

	V469_17490	fecC	3837373	3838392	1020	ABC-type Fe3+-siderophore transport system, permease component
	V469_17495	fecE	3838389	3839129	741	ABC-type Fe3+-siderophore transport system, ATP-binding protein
ML09-119	AHML_05675	fecA_1	1251210	1249090	2121	TonB-dependent receptor
	AHML_05670	pdtP	1249087	1248017	1071	Methyltransferase type 12
	AHML_05665	ssuA	1248020	1246992	1029	ABC-type nitrate/sulfonate/bicarbonate transport system. substrate-binding protein
	AHML_05660	ssuC	1247005	1246253	753	ABC-type nitrate/sulfonate/bicarbonate ABC transport system, permease protein
	AHML_05655	ssuB	1246007	1245582	426	ABC-type nitrate/sulfonate/bicarbonate transport system, ATP-binding protein
	AHML_05650	norR	1245368	1244895	474	Anaerobic nitric oxide reductase transcription regulator
	AHML_05645	fecA_2	1244785	1242746	2040	TonB-dependent receptor
	AHML_05640	fnr	1241975	1242691	717	Fumarate and nitrate reduction regulatory protein
	AHML_05635	fecB	1241788	1240799	990	ABC-type Fe3+-siderophore transport system, substrate-binding protein
	AHML_05630	fecC	1240799	1239780	1020	ABC-type Fe3+-siderophore transport system, permease component
	AHML_05625	fecE	1239783	1239043	741	ABC-type Fe3+-siderophore transport system, ATP-binding protein
AL09-71	V428_05900	fecA_1	1250834	1248762	2073	TonB-dependent receptor
	V428_05895	pdtP	1248759	1247689	1071	Methyltransferase type 12
	V428_05890	ssuA	1247692	1246664	1029	ABC-type nitrate/sulfonate/bicarbonate transport system. substrate-binding protein
	V428_05885	ssuC	1246677	1245925	753	ABC-type nitrate/sulfonate/bicarbonate ABC transport system, permease protein
	V428_05880	ssuB	1245928	1245254	675	ABC-type nitrate/sulfonate/bicarbonate transport system, ATP-binding protein
	V428_05875	norR	1245040	1244567	474	Anaerobic nitric oxide reductase transcription regulator
	V428_05870	fecA_2	1244457	1242418	2040	TonB-dependent receptor
	V428_05865	fnr	1241647	1242363	717	Fumarate and nitrate reduction regulatory protein
	V428_05860	fecB	1241550	1240471	1080	ABC-type Fe3+-siderophore transport system, substrate-binding protein
	V428_05855	fecC	1240471	1239452	1020	ABC-type Fe3+-siderophore transport system, permease component
	V428_05850	fecE	1239455	1238715	741	ABC-type Fe3+-siderophore transport system, ATP-binding protein
pc104A	V429_05900	fecA_1	1250833	1248761	2073	TonB-dependent receptor
	V429_05895	pdtP	1248773	1247688	1086	Methyltransferase type 12
	V429_05890	ssuA	1247691	1246663	1029	ABC-type nitrate/sulfonate/bicarbonate transport system. substrate-binding protein
	V429_05885	ssuC	1246676	1245924	753	ABC-type nitrate/sulfonate/bicarbonate ABC transport system, permease protein
	V429_05880	ssuB	1245927	1245253	675	ABC-type nitrate/sulfonate/bicarbonate transport system, ATP-binding protein
	V429_05875	norR	1245039	1244566	474	Anaerobic nitric oxide reductase transcription regulator
	V429_05870	fecA_2	1244456	1242417	2040	TonB-dependent receptor
	V429_05865	fnr	1241646	1242362	717	Fumarate and nitrate reduction regulatory protein
	V429_05860	fecB	1241549	1240470	1080	ABC-type Fe3+-siderophore transport system, substrate-binding protein
	V429_05855	fecC	1240470	1239451	1020	ABC-type Fe3+-siderophore transport system, permease component
	V429_05850	fecE	1239454	1238714	741	ABC-type Fe3+-siderophore transport system, ATP-binding protein

Table S8. *A. hydrophila* strains of ST251 clonal group.

Strains	Biological source	Geographic source	Date of isolation	MLST (ST)	Allele						Reference or accession number
					gyrB	*groL*	*gltA*	*metG*	*ppsA*	*recA*	
ZC1	Grass carp	Guangdong, China	UD[a] (before Dec, 2008)	251	210	214	122	211	221	217	6
XX-14	Silver carp	Henan, China	2010	328	210	214	122	211	271	217	This study
XX-22	Common carp	Henan, China	2010	251	210	214	122	211	221	217	This study
XX-49	Crucian carp	Henan, China	2010	251	210	214	122	211	221	217	This study
XX-52	Silver carp	Henan, China	2010	328	210	214	122	211	271	217	This study
XX-58	Silver carp	Henan, China	2010	251	210	214	122	211	221	217	This study
XX-62	Crucian carp	Henan, China	2010	251	210	214	122	211	221	217	This study
XS91-4-1	Silver loweye carp	Hubei, China	1991	251	210	214	122	211	221	217	7
IB101	Wuchang bream	Hubei, China	2006	251	210	214	122	211	221	217	7
IB336	Wuchang bream	Hubei, China	2006	251	210	214	122	211	221	217	7
4LNG101	Silver loweye carp	Hubei, China	2008	251	210	214	122	211	221	217	7
4LNG201	Silver loweye carp	Hubei, China	2008	251	210	214	122	211	221	217	7
4LNS301	Silver loweye carp	Hubei, China	2008	251	210	214	122	211	221	217	7
JG101	Crucian carp	Hubei, China	2008	251	210	214	122	211	221	217	7
CG101	Grass carp	Hubei, China	2008	251	210	214	122	211	221	217	7
LNB101	Silver loweye carp	Hubei, China	2008	251	210	214	122	211	221	217	7
DWCG101	Wuchang bream	Hubei, China	2009	251	210	214	122	211	221	217	7
DLNG101	Silver loweye carp	Hubei, China	2009	251	210	214	122	211	221	217	7
DLNG201	Silver loweye carp	Hubei, China	2009	251	210	214	122	211	221	217	7
DBHS101	Bighead carp	Hubei, China	2009	251	210	214	122	211	221	217	7
JBN1001	Crucian carp	Hubei, China	2009	251	210	214	122	211	221	217	7
JBN1101	Crucian carp	Hubei, China	2009	251	210	214	122	211	221	217	7
JBN1201	Crucian carp	Hubei, China	2009	251	210	214	122	211	221	217	7
JBN1301	Crucian carp	Hubei, China	2009	251	210	214	122	211	221	217	7
2JBN101	Crucian carp	Hubei, China	2009	251	210	214	122	211	221	217	7
2JBN301	Crucian carp	Hubei, China	2009	251	210	214	122	211	221	217	7
2JFN201	Crucian carp	Hubei, China	2009	251	210	214	122	211	221	217	7
2WCL101	Wuchang bream	Hubei, China	2009	251	210	214	122	211	221	217	7
NSC90-4-1	Silver loweye carp	Hunan, China	1990	251	210	214	122	211	221	217	7
J-1	Crucian carp	Jiangsu, China	1989	251	210	214	122	211	221	217	This study
NJ-1	Crucian carp	Jiangsu, China	2008	251	210	214	122	211	221	217	This study
NJ-34	Crucian carp	Jiangsu, China	2010	251	210	214	122	211	221	217	This study
NJ-35	Crucian carp	Jiangsu, China	2010	251	210	214	122	211	221	217	This study
SQ13-11	Water	Jiangsu, China	2013	251	210	214	122	211	221	217	This study
DW14-1	Diseased crucian carp	Jiangsu, China	2014	251	210	214	122	211	221	217	This study
JD14-1	Diseased crucian carp	Jiangsu, China	2014	251	210	214	122	211	221	217	This study
ML14-9	Diseased silver carp	Jiangsu, China	2014	251	210	214	122	211	221	217	This study
XS14-1	Diseased bream	Jiangsu, China	2014	251	210	214	122	211	221	217	This study
BSK-10	Crucian carp	Zhejiang, China	UD (before Sep, 2001)	UD	210	214	122	211	221	217	This study
ML09-119	Channel catfish	Alabama, USA	2009	251	210	214	122	211	221	217	8
AL09-71	Channel catfish	Alabama, USA	2009	251	210	214	122	211	221	217	9
AL09-79	Channel catfish	Alabama, USA	2009	251	210	214	122	211	221	217	8
PC104A	Soil of catfish pond	Alabama, USA	2010	251	210	214	122	211	221	217	10
PB10-118	Channel catfish	Arkansas, USA	2010	251	210	214	122	211	221	217	8
S04-690	Channel catfish	Mississippi, USA	2004	251	210	214	122	211	221	217	8

[a] UD represents "undetermined".

Table S9. Primers used in this study.

Target gene	Primer	Sequence (5' to 3')	Size of PCR amplicon (bp)	Annealing temp (℃)	Size of sequence used in MLST (bp)	Reference
gyrB	*gyrB*_F	GGGGTCTACTGCTTCACCAA	669	59	477	11
	*gyrB*_R	CTTGTCCGGGTTGTACTCGT				
groL	*groL*_F	CAAGGAAGTTGCTTCCAAGG	782	56	510	11
	*groL*_R	CATCGATGATGGTGGTGTTC				
gltA	*gltA*_F	TTCCGTCTGCTCTCCAAGAT	626	58	495	11
	*gltA*_R	GAAGATCACGGTGAACATGG				
metG	*metG*_F	TGGCAACTGATCCTCGTACA	657	57	504	11
	*metG*_R	TCTTGTTGGCCATCTCTTCC				
ppsA	*ppsA*_F	AGTCCAACGAGTACGCCAAC	619	60	537	11
	*ppsA*_R	TCGGCCAGATAGAGCCAGGT				
recA	*recA*_F	AGAACAAACAGAAGGCACTGG	640	57	561	11
	*recA*_R	AACTTGAGCGCGTTACCAC				
T2SS	*exeN*_F	GCTGATTGCCGTGCTGTTTC	169	60		This study
	*exeN*_R	CGACTCGCTGGCGTATTGC				
	*exeB*_F	GAAGACGGTGGAGGTGAAGG	195	55		This study
	*exeB*_R	GTGCTGTTGAGGGCATTGAG				
	*tapD*_F	CAGCAGGAAGAACGCTACAA	573	55		This study
	*tapD*_R	AATGAGACCAATCCCGATGA				
T3SS	*ascV*_F	AGCAGATGAGTATCGACGG	891	58		12
	*ascV*_R	AGGCAT TCTCCTGTACCAG				
	*aopB*_F	TACCTGTTGGAATGATTCCG	951	58		12
	*aopB*_R	AGTGAACGCCCTCTCTCC				
	*aexT*_F	CTGGAGGCTCGTCAGGTTG	376	54		This study
	*aexT*_R	TCCCGTTCAGTGAGGTTGC				
	*aexU*_F	GTTGGCCCGTCCGTTTGT	440	56		This study
	*aexU*_R	GCTGGCTCATTGCCTCTTGT				
T6SS	*vasH*_F	TGGCGGTGGACGATTTTT	353	57		This study
	*vasH*_R	CTTCATCTGACGCAGGGAGT				
	*hcp*_F	CTGCTGGTCAGCGTGTCC	195	57		This study
	*hcp*_R	CGATGTCAACGATGGTGGC				
	*vgrG*_F	GAGGCGTAAATGGCAGACA	497	55		This study
	*vgrG*_R	GCGGCGAGTCGGTTCA				
Polar flagella	*flhA*_F	TGCCTCGAAATTCGTGAAA	539	52		This study
	*flhA*_R	CCAGCGGGATGAGTTGATA				
	*flgA*_F	GCAATACCACCGTCTACCTC	428	50		This study
	*flgA*_R	GCCAACAGCGTTGACTTTA				
Lateral flagella	*flgL*_F	AAGCAAGACATTACAGGACGAT	160	55		This study
	*flgl*_R	GGTGACGGCACTGGCATA				
	*lafB*_F	CAAGGATAGTGGGTTGGAG	619	55		13
	*lafB*_R	CAAAGCACCATCGCGTTTG				
	*lafT*_F	TGGTGTTTCATGATGGCGG	671	55		13
	*lafT*_R	ATCAGCAGCCCTATTC GAC				
Toxin	*aerA*_F	GCTGGTCAAGACGGTGGT	255	50		This study
	*aerA*_R	GATTGCGACAGGGAGGTG				
	*ahh1*_F	GGGAGTGGAAAGCGGAC	155	52		This study
	*ahh1*_R	GAAGATGCGGGTGTAGGAG				
	*ast*_F	CCTATGTCGGCACCTTTG	372	50		This study
	*ast*_R	CATCCCTTGGCTTGTTGT				
Enzyme	*ahpB*_F	GTGATGCAGGATGAGGC	306	48		This study
	*ahpB*_R	GGTGAACTGATGAATGGC				
	*eprA1*_F	TCCCATTGCCCTGTTGCT	462	55		This study
	*eprA1*_R	TCACTCACTCCGTCCACCC				
	*plc*_F	CACCGATGTCTACCTCACCAA	692	55		This study
	*plc*_R	AGCGGCTGACGATTACCAC				
	*ser*_F	CTACGGCTTTGGTCTCATCG	394	55		This study
	*ser*_R	CGCTTCGCCATAGAACTTGT				
myo-inositol utilization pathway	*iolA*_F	CATTGGCGTTACGATTGG	560	55		This study
	*iolA*_R	GAAGAAGCCCTGTTTGTGAG				
	*iolG2*_F	GGTCGGATTGGTCAGGTTC	207	52		This study
	*iolG2*_R	AATGAGATCGGCATGGGTAT				
	*iolE*_F	GCAGCGTGGAAGAGGAGAT	402	52		This study
	*iolE*_R	CTTTGCAGTGAACGTGGTTG				
	*rbsA*_F	ATGTCGCAACCCTTACTGA	234	54		This study
	*rbsA*_R	TCAGTAAGGGTTGCGACAT				
L-fucose utilization pathway	*nanA*_F	ATTGGTCCTTGCTTCTTCA	417	50		This study
	*nanA*_R	TTCGATCACGCTGTTCATA				
	*nanE*_F	TTACGCCGTATCTGGAGGA	299	55		This study
	*nanE*_R	CGCCATTACAAAGCACCC				
Sialic acid uitlization pathway	*fucP*_F	CCGTATGTAGTGGTGGCG	650	50		This study
	*fucP*_R	CCTTTGAAACCGAAGTGGA				
	*fucK*_F	TGGCGGTGATGGAGCAAG	474	57		This study
	*fucK*_R	TTTCCCAGGTCCCGGATG				

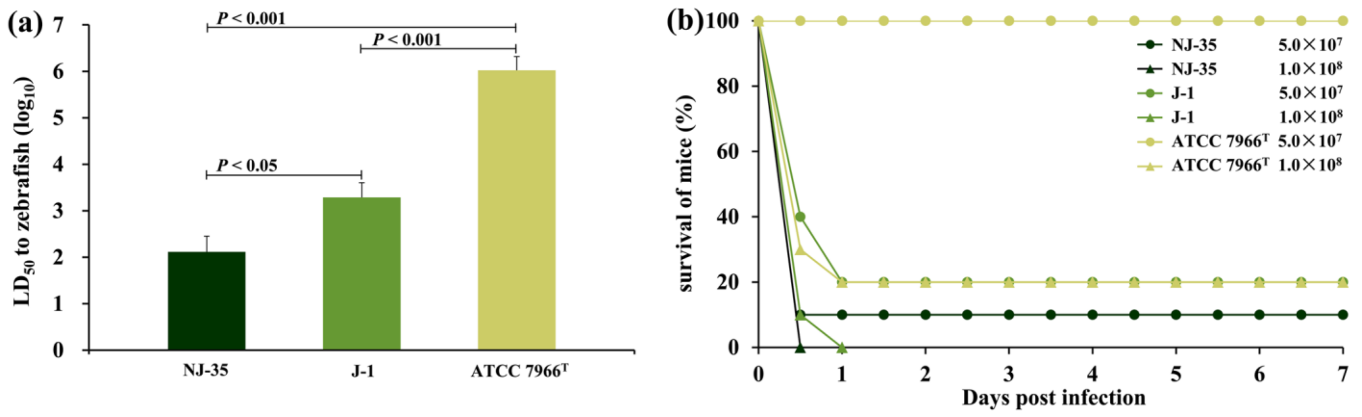

Figure S1. Virulence of *A. hydrophila* strains assessed in zebrafish (a) and ICR mice (b). The virulence of *A. hydrophila* strains are depicted using LD_{50}s in zebrafish and survival of ICR mice, respectively. Statistical significance was analyzed by one-way analysis of variance (ANOVA), followed by Turkey's multiple comparison test.

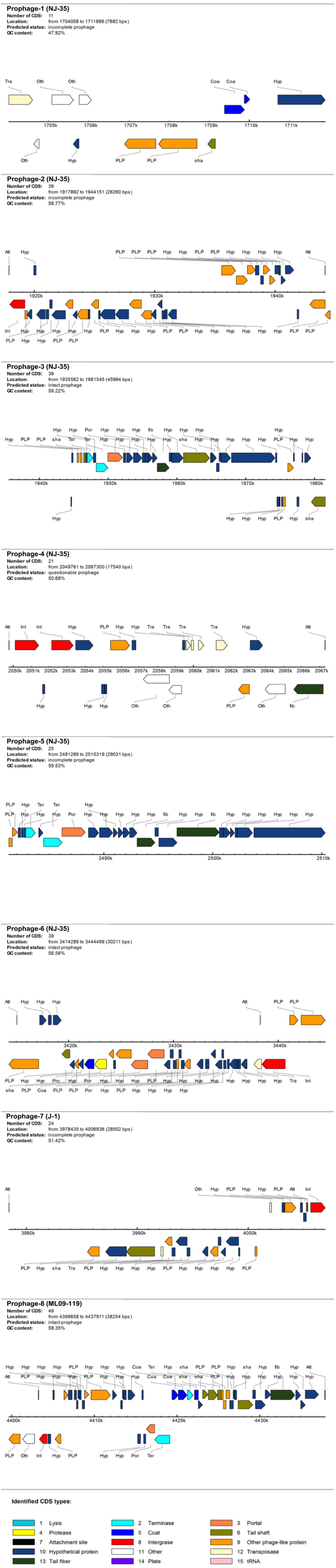

Figure S2. Characterization of the prophage regions predicted in *A. hydrophila* NJ-35, J-1 and ML09-119. These prophage regions were predicted with PHAST.

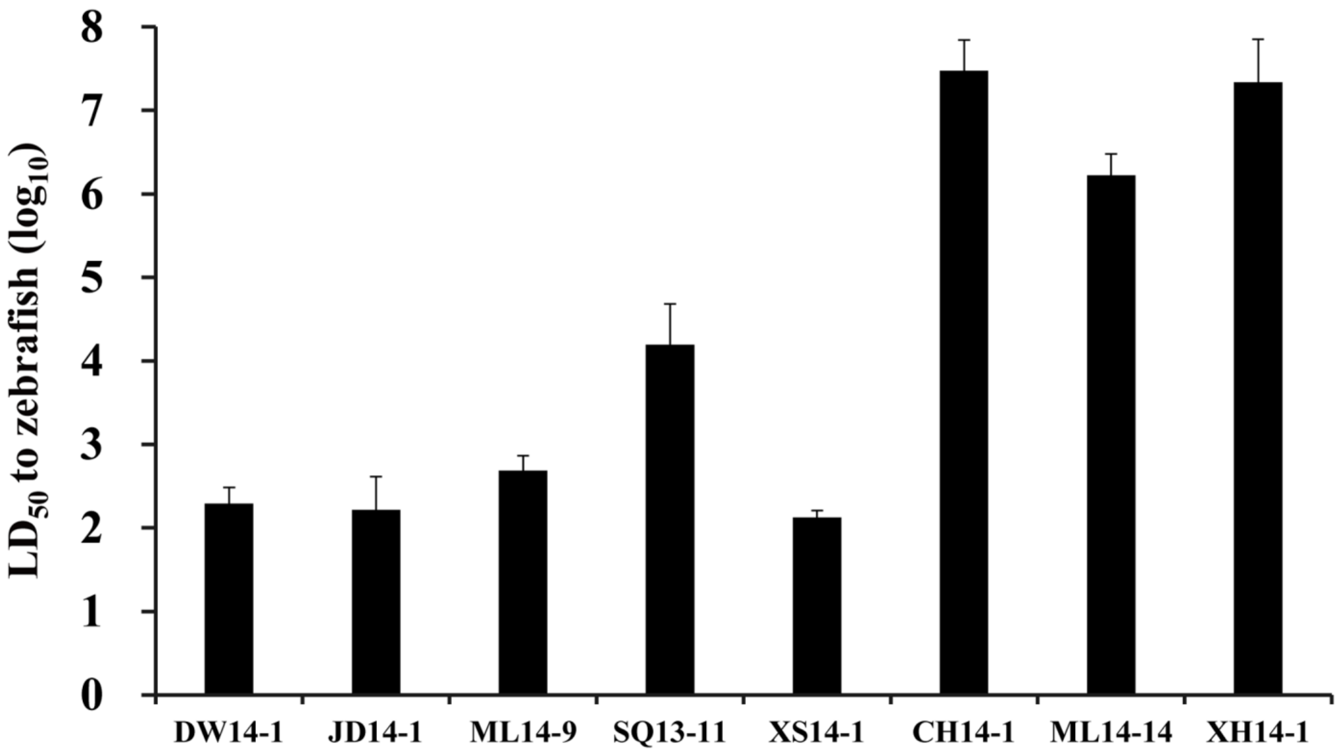

Figure S3. Virulence of *A. hydrophila* strains assessed in zebrafish. The virulence of *A. hydrophila* strains are depicted using LD_{50}s in zebrafish.

References

1. Sandkvist M. Type II secretion and pathogenesis. *Infect Immun* **69**, 3523-3535 (2001).

2. Canals R, *et al.* Polar flagellum biogenesis in *Aeromonas hydrophila*. *J Bacteriol* **188**, 542-555 (2006).

3. Boyd JM, *et al.* Contribution of type IV pili to the virulence of *Aeromonas salmonicida* subsp. *salmonicida* in Atlantic salmon (*Salmo salar* L.). *Infect Immun* **76**, 1445-1455 (2008).

4. Ebanks RO, Dacanay A, Goguen M, Pinto DM, Ross NW. Differential proteomic analysis of *Aeromonas salmonicida* outer membrane proteins in response to low iron and in vivo growth conditions. *Proteomics* **4**, 1074-1085 (2004).

5. Richter M, Rossello-Mora R. Shifting the genomic gold standard for the prokaryotic species definition. *Proc Natl Acad Sci U S A* **106**, 19126-19131 (2009).

6. Deng GC, *et al.* Isolation, identification and characterization of *Aeromonas hydrophila* from hemorrhagic grass carp. *Microbiol China* **36**, 1170–1177. (2009).

7. Zhang X, Yang W, Wu H, Gong X, Li A. Multilocus sequence typing revealed a clonal lineage of *Aeromonas hydrophila* caused motile *Aeromonas* septicemia outbreaks in pond-cultured cyprinid fish in an epidemic area in central China. *Aquaculture* **432**, 1-6 (2014).

8. Hossain MJ, *et al.* An Asian origin of virulent *Aeromonas hydrophila* responsible for disease epidemics in United States-farmed catfish. *mBio* **5**, e00848-00814 (2014).

9. Pridgeon JW, Klesius PH. Virulence of *Aeromonas hydrophila* to channel catfish Ictaluras punctatus fingerlings in the presence and absence of bacterial extracellular products. *Dis Aquat Organ* **95**, 209-215 (2011).

10. Griffin MJ, *et al.* Rapid quantitative detection of *Aeromonas hydrophila* strains associated with disease outbreaks in catfish aquaculture. *J Vet Diagn Invest* **25**, 473-481 (2013).

11. Martino ME, Fasolato L, Montemurro F, Novelli E, Cardazzo B. *Aeromonas* spp.: ubiquitous or specialized bugs? *Environ Microbiol* **16**, 1005-1018 (2014).

12. Carvalho-Castro GA, Lopes CO, Leal CA, Cardoso PG, Leite RC, Figueiredo HC. Detection of type III secretion system genes in *Aeromonas hydrophila* and their relationship with virulence in Nile tilapia. *Vet Microbiol* **144**, 371-376 (2010).

13. Gavin R, Rabaan AA, Merino S, Tomas JM, Gryllos I, Shaw JG. Lateral flagella of Aeromonas species are essential for epithelial cell adherence and biofilm formation. *Mol Microbiol* **43**, 383-397 (2002).
